# Benzodiazepine receptor agonists in hospitalised patients in the Netherlands: initiation, continuation and discontinuation – a retrospective observational analysis

**DOI:** 10.1136/bmjopen-2025-112758

**Published:** 2026-02-09

**Authors:** Carlijn J de Gans, Eva S van den Ende, Arjen J G Meewisse, Mark L van Zuylen, Dirk Jan Stenvers, Jeroen Hermanides, Prabath W B Nanayakkara

**Affiliations:** 1 Department of Internal Medicine, Section General Internal Medicine unit Acute Medicine, Amsterdam University Medical Center, Amsterdam, The Netherlands; 2 Amsterdam Public Health Research Institute, Quality of Care, Amsterdam University Medical Centre, Vrije Universiteit Amsterdam, Amsterdam, The Netherlands; 3 Amsterdam Public Health Research Institute, Quality of Care, Amsterdam University Medical Centre, University of Amsterdam, Amsterdam, The Netherlands; 4 Department of Anesthesiology, Amsterdam University Medical Center, Amsterdam, The Netherlands; 5 Department of Pediatric Intensive Care, Amsterdam University Medical Center, Amsterdam, The Netherlands; 6 Department of Endocrinology and Metabolism, Amsterdam University Medical Center, Amsterdam, The Netherlands; 7 Amsterdam Gastroenterology Endocrinology and Metabolism (AGEM), Amsterdam University Medical center, Amsterdam, The Netherlands

**Keywords:** Quality Improvement, SLEEP MEDICINE, Hospitals, GENERAL MEDICINE (see Internal Medicine)

## Abstract

**Objective:**

To examine inpatient benzodiazepine receptor agonists prescribing patterns and assess how hospitalisation affects use at discharge.

**Design:**

Subanalysis of the WEsleep trial, a cluster-randomised controlled single-centre study conducted at Amsterdam University Medical Center (Amsterdam UMC) (two locations) between July 2023 and March 2024. Twelve departments (six medical, six surgical) were matched and randomised to intervention or standard care. On intervention wards, multiple measures to improve sleep were implemented, including minimising nighttime disruptions.

**Setting:**

Amsterdam UMC, across medical and surgical hospital departments.

**Patients:**

Adult patients admitted for ≥2 nights (medical) or undergoing elective non-cardiac surgery in a surgical department.

**Primary and secondary outcome measures:**

Benzodiazepine use was classified as no use, pre-admission use or new in-hospital initiation. Prescribing patterns were summarised descriptively according to type, timing, indication and discharge status.

**Results:**

Of 746 patients, 187 (25%) used benzodiazepines: 80 (43%) had pre-admission use, and 107 (57%) were newly initiated during their hospital stay. Among pre-admission users, two discontinued and five had adjustments at discharge. Among newly initiated users, 94 (88%) had their benzodiazepine discontinued at discharge. Approximately half of pre-admission prescriptions and one-third of in-hospital prescriptions lacked a documented indication.

**Conclusions:**

Although most newly initiated benzodiazepine treatments were discontinued during hospitalisation, pre-existing use was rarely reassessed and nearly 10% of new users were discharged with a prescription. Structured deprescribing protocols, better documentation of indications and improved discharge planning are needed to promote safer and more rational benzodiazepine use.

**Trial registration number:**

NCT05683483.

**STRENGTHS AND LIMITATIONS OF THIS STUDY**

- Data were derived from a cluster-randomised controlled trial, ensuring a robust methodological framework.
- Benzodiazepine prescribing was systematically captured across pre-admission, in-hospital and discharge phases.
- A pragmatic approach to classify daytime versus evening use enabled consistent analysis, though minor misclassification may have occurred.
- Broad inclusion and exclusion criteria allowed enrolment of a heterogeneous hospital population, supporting generalisability of findings.

## Introduction

 Benzodiazepine receptor agonists are among the most commonly prescribed psychotropic medications worldwide, indicated primarily for the short-term treatment of anxiety, insomnia and acute agitation. They are also used as muscle relaxants and anticonvulsants.^1^ Despite their clinical usefulness in acute situations, such as during hospital admissions, benzodiazepines are often used for prolonged periods, contrary to guideline recommendations.^2^ Long-term use is associated with well-documented adverse effects, including tolerance, physical and psychological dependence, cognitive impairment and an increased risk of falls, particularly among older adults.^3^,^5^ Hospitalisation represents a critical moment in a patient’s treatment trajectory, where medications are frequently initiated, adjusted or discontinued.^6^

In line with global trends, the use of sleep and sedative medications remains common in the Netherlands. According to data from the National Drug Monitor, 18.9% of adults aged 18 years and older reported having used such medications at some point in their lives, and 9.6% reported use in the past year.^7^ These figures underscore the continued and widespread reliance on benzodiazepines and related agents in the general population.

However, it is unclear to what extent hospital admissions contribute to the initiation or continuation of benzodiazepine use. On the one hand, hospital stays may offer an opportunity to stop inappropriate benzodiazepine prescriptions initiated in the primary care setting.^8^ On the other hand, the combination of poor sleep quality during hospitalisation due to external factors such as noise and interruptions, and internal factors like illness, pain or surgery,^9 10^ and the overall stress of admission may prompt new benzodiazepine prescriptions for insomnia or anxiety, with potential for continuation after discharge. Previous studies have reported that between 22% and 33% of hospitalised patients receive benzodiazepines during their stay, underscoring their frequent use in inpatient care.^11 12^ Prior qualitative studies have also identified key barriers to inpatient benzodiazepine deprescribing that may contribute to the continued prescribing of these medications.^13^

This study examines benzodiazepine prescribing patterns in hospitalised medical and surgical patients by comparing patients who used benzodiazepine receptor agonists prior to admission with those who were newly initiated during hospitalisation. The aim is to describe benzodiazepine receptor agonist use during hospital stay and at discharge and to explore the potential contribution of hospital prescribing practices to benzodiazepine use at the time of discharge.

## Methods

### Study design

This study is a subanalysis of data derived from the WEsleep trial, a cluster-randomised controlled single-centre study conducted at Amsterdam University Medical Center (Amsterdam UMC), including two locations. In brief, the primary aim of the WEsleep trial was to improve sleep during hospitalisation; secondary outcomes included sleep quantity, use of sleep medication, timing of diuretics and corticosteroids, length of admission, delirium incidence and 30-day mortality. A detailed description of the main trial is provided elsewhere^14^ (for study protocol, see online supplemental file 2). The study adheres to the Consolidated Standards of Reporting Trials guidelines and is registered on ClinicalTrials.gov (NCT05683483).

The present subanalysis specifically examines benzodiazepine and benzodiazepine-related drug (Z-drug) prescribing patterns during hospitalisation and at discharge.

We analysed medication use at admission, throughout the hospital stay and at discharge, distinguishing between patients with pre-existing benzodiazepine/Z-drug use and those who initiated use during hospitalisation. The primary focus was on initiation, continuation, discontinuation, daily use during admission and documentation of prescribing indications.

### Patients

This subanalysis of data from the WEsleep trial includes both medical and surgical patients. Although the two groups were recruited under distinct inclusion criteria, both were part of the same overarching study and are jointly analysed in the present work. Patients were eligible if they were 18 years of age or older, expected to remain hospitalised for at least two nights (for medical patients) and capable of providing written informed consent. Surgical patients were only eligible if they would undergo elective non-cardiac surgery with at least one planned overnight postoperative hospital stay. Patients were excluded if they were placed in strict or airborne infection isolation, were unable to communicate in Dutch, had admission to the intensive care unit (planned or unplanned), had pre-existing cognitive dysfunction or presented with active delirium at the time of inclusion, and for surgical patients, had an American Society of Anesthesiologists physical status classification of 4 or higher.

### Data collection

For the present analysis, data were extracted from the WEsleep database focusing on benzodiazepine receptor agonist use.

For each patient, benzodiazepine receptor agonists use was recorded at hospital admission (home medication), on each day during hospitalisation and at discharge. For every hospital day, it was documented whether a benzodiazepine receptor agonist was administered and, if so, which agent was used. In addition to classical benzodiazepines, benzodiazepine-related drugs (Z-drugs), including zolpidem and zopiclone, were included in the analyses because of their similar mechanism of action and comparable clinical effects, adverse event profiles and risks associated with prolonged use.

Information on pre-admission benzodiazepine receptor agonist use was obtained through structured medication reconciliation performed by the hospital pharmacy as part of routine care, based on community pharmacy dispensing records and patient interviews. Discharge medication data were retrieved from the hospital prescribing system and discharge letters. Prescribing indications were obtained from documentation in the hospital medication system or, for home medication, from the medication reconciliation records.

A distinction was made between daytime and evening use. For home medication, this classification was based on prescription instructions. If the prescription explicitly stated use at night, it was classified as evening medication; if no such specification was provided, it was considered daytime medication. For in-hospital use, medications prescribed or administered between 16:00 and 06:00 were classified as evening medication.

At hospital discharge, discontinuation of benzodiazepines was implemented in the hospital prescribing system and communicated electronically to the community pharmacy, and medication changes were documented in the discharge letter to the general practitioner.

### Statistical analysis

Descriptive statistics were used to characterise the study population. Patients were categorised into three groups based on benzodiazepine use: no use, pre-admission use and new in-hospital initiation. Baseline characteristics were described for each group, including age, sex, body mass index, history of intoxication, comorbidities, hospital department and whether patients were admitted for surgical or non-surgical reasons. For continuous variables, the distribution was assessed using histograms and Q-Q plots. Depending on normality, either the mean with SD or the median with IQR was reported. For categorical variables, absolute numbers and percentages were presented.

In addition, benzodiazepine-related prescribing patterns were explored, including the type of benzodiazepine used, indication for prescription, timing (daytime or evening) and any changes during the hospital stay or at discharge.

All statistical analyses were conducted using IBM SPSS Statistics, V. 28.

### Patient and public involvement

Patients or members of the public were not involved in the design, conduct, reporting or dissemination of the current subanalysis. The present study is based on previously collected data from the main study, and no additional patient involvement was required for this secondary work.

## Results

**Figure 1:**
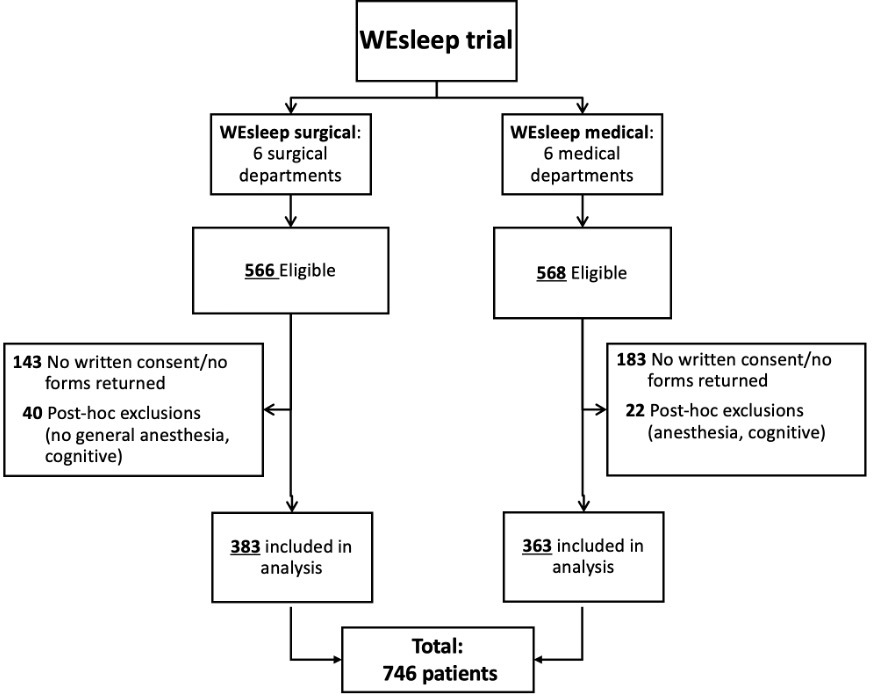
Flowchart.

A total of 746 patients were included from the WEsleep trial dataset (figure 1). Baseline characteristics of the study population are presented in table 1. Overall, 80 patients used benzodiazepines prior to hospital admission, and 107 patients were newly initiated on benzodiazepines during hospitalisation.

**Table 1:** Baseline characteristics of the included patients, categorised in use of benzodiazepines

	No prescribed benzodiazepines (n=559)	Benzodiazepines prescribed at home (n=80)	Benzodiazepines newly initiated in the hospital (n=107)
Age, median (IQR)	59.0 (46.0–69.0)	61.0 (55.3–67.0)	62.0 (47.0–70)
<50 years, n (%)	164 (29.3)	12 (15.0)	31 (29.0)
50–70 years, n (%)	276 (49.4)	55 (68.8)	50 (46.7)
>70 years, n (%)	119 (21.3)	13 (16.3)	26 (24.3)
Sex			
Male, n (%)	294 (52.6)	27 (33.8)	43 (40.2)
Female, n (%)	264 (47.2)	53 (66.3)	64 (59.8)
Other, n (%)	1 (0.2)		
Body mass index, median (IQR)	25.3 (22.3–29.4)	24.4 (21.4–27.6)	25.1 (22.4–27.7)
Missing body mass index, n (%)	75 (13.4)	6 (7.5)	8 (7.5)
Comorbidities			
No/blank medical history	17 (3.0)	0 (0.0)	1 (0.9)
≤2 comorbidities	191 (34.2)	21 (26.3)	32 (29.9)
3–5 comorbidities	233 (41.7)	40 (50.0)	56 (52.3)
≥6 comorbidities	118 (21.1)	19 (23.8)	18 (16.8)
Type of patient			
Surgical	270 (48.3)	39 (48.8)	74 (69.2)
Non-surgical/medical	289 (51.7)	41 (51.2)	33 (30.8)
Location Amsterdam UMC (AMC or VUmc)			
AMC, n (%)	277 (49.6)	33 (41.3)	48 (44.9)
VUmc, n (%)	282 (50.4)	47 (58.8)	59 (55.1)
Intervention/standard care WEsleep trial			
Intervention	295 (52.8)	40 (50)	45 (42.1)
Standard care	264 (47.2)	40 (50)	62 (57.9)
30-day mortality, n (%)	5 (0.9)	2 (2.5)	1 (0.9)
Intensive care unit admission, yes, n (%)	5 (0.9)	0 (0.0)	1 (0.9)
Length of stay in days, median (IQR)	3 (2–5)	3 (2–5)	4 (2–6)
Missing, n (%)	15 (2.7)	1 (1.3)	2 (1.9)

Among the 80 patients who used benzodiazepines prior to hospital admission, the majority were between 50 and 70 years of age (68.8%), with fewer patients younger than 50 or older than 70 years. Most patients in this group were female (66.3%, n=54). These patients generally continued benzodiazepine use during hospitalisation.

Of the 107 patients who were newly initiated on benzodiazepines during hospitalisation, initiation occurred more frequently in surgical than in non-surgical patients, with 69.2% (n=74) admitted for surgical care. Women were also over-represented in this group, comprising 59.8% (n=64) of newly treated patients.

### Benzodiazepine use at home

For benzodiazepine use at home, the most commonly prescribed benzodiazepines during the daytime were oxazepam (5–25 mg), lorazepam (0.5–1 mg) and diazepam (5–10 mg) (online supplemental table 1). In the evening, temazepam (10–20 mg), zolpidem (5–10 mg) and zopiclone (3.75–15 mg) were most frequently prescribed (online supplemental table 2). In 41 of 80 patients, the indication for use was known. The most common indications for benzodiazepine use at home were insomnia (15 out of 80 patients; 19%) and anxiety (12 out of 80; 15%). Other reported indications included a combination of symptoms, nausea, stress/tension and alcohol abuse (table 2).

**Table 2:** Indications for benzodiazepine prescriptions before and during hospitalisation

Indication	Pre-admission (n=80)	During admission (n=107)
Insomnia	15 (18.8%)	32 (29.9%)
Anxiety	12 (15.0%)	9 (8.4%)
Insomnia and anxiety	4 (5.0%)	1 (0.9%)
Anxiety and tension	–	2 (1.9%)
Tension	6 (7.5%)	–
Nausea	3 (3.8%)	1 (0.9%)
Premedication/evening medication for anaesthesia	–	28 (26.2%)
As-needed for potential status epilepticus	–	1 (0.9%)
Alcohol abuse	1 (1.3%)	–
Unknown	39 (48.8%)	33 (30.8%)

Within this subgroup, 39 patients (48.8%) were surgical and 41 patients (51.2%) were medical. In 11 patients (14%), a change in benzodiazepine medication occurred during hospitalisation; in six (7.5%) patients, the medication was not prescribed during admission (for unclear reasons, and with uncertainty as to whether the patient used it independently), while in five patients (6.3%), the type, dosage or frequency of the benzodiazepine was modified.

Upon discharge, a change compared with the home medication at admission was made in seven patients (8.8%) and in another seven (8.8%), the status was unknown. In two patients, benzodiazepines were discontinued, while in five patients, a change was made in the type or frequency of the benzodiazepine.

### Benzodiazepines newly initiated during hospital admission

Oxazepam 10 mg was the most frequently prescribed benzodiazepine during daytime hours. Midazolam 7.5 mg was prescribed less often, followed by single recorded prescriptions for midazolam 1 mg intravenously, diazepam 10 mg and lorazepam 1 mg (online supplemental table 3).

During evening hours, temazepam 10 mg was the most frequently prescribed benzodiazepine, followed by oxazepam in doses of 5–10 mg. Lorazepam (0.5–2 mg) was used less frequently, while zolpidem (5–10 mg), zopiclone (7.5 mg) and midazolam (7.5 mg) were each prescribed or administered occasionally. Diazepam (2–10 mg) was used least frequently during the evening period (online supplemental table 4).

Benzodiazepines were more frequently prescribed to surgical patients (74 patients; 69.2%) than to medical patients (33 patients; 30.8%). The three most common indications for benzodiazepine use during hospitalisation were insomnia, premedication/evening medication related to anaesthesia and anxiety. The indication was unknown for 33 (31%) out of 107 patients (table 2).

In eight patients (7.5%), a benzodiazepine was prescribed at discharge despite no documented use prior to admission. For five patients, benzodiazepine use at discharge was unknown. In 94 out of 107 patients (88%), the newly started benzodiazepine was discontinued upon discharge. Among the eight patients discharged with a new benzodiazepine, lorazepam was prescribed three times, temazepam four times and oxazepam once.

## Discussion

This study investigated benzodiazepine prescribing patterns in hospitalised patients, focusing on those with pre-existing use at home and those newly initiated during admission, to evaluate how hospitalisation affects usage at discharge. Among 80 patients with prior benzodiazepine use, in only seven a medication change was initiated during hospital stay. Of the 107 patients who were started on benzodiazepines during hospitalisation, the medication was discontinued at discharge in 94 cases.

These findings point to two key observations. First, benzodiazepines started during hospitalisation were generally intended for short-term use, as reflected by the high discontinuation rate at discharge. Second, in patients already using benzodiazepines prior to admission, hospital stays rarely resulted in modification or discontinuation of therapy. This limited reassessment was further underscored by the frequent absence of documented prescribing indications in both groups. Although undocumented indications do not necessarily imply the absence of clinical reasoning, they limit opportunities for critical evaluation and formal reassessment during admission.

Despite the generally cautious approach to newly initiated benzodiazepine use, nearly 1 in 10 patients without prior use were still discharged with a new prescription. Compared with previous observational research reporting continued use after discharge in less than 1% of patients,^15^ this difference may partly reflect differences in outcome assessment, as our study evaluated prescribing at hospital discharge, whereas the referenced study assessed subsequent prescribing in primary care. Nevertheless, our findings suggest that a meaningful proportion of new inpatient prescriptions may extend beyond the hospital setting. In addition, positive experiences with benzodiazepines during hospitalisation may influence patients’ expectations after discharge and contribute to subsequent requests for these medications in outpatient care.^16^

In addition to differences in prescribing patterns, subgroup differences were observed in surgical admission and sex distribution. Benzodiazepine initiation occurred more frequently among surgical patients, which may relate to perioperative use or differences in prescribing culture between surgical and medical departments.^17^ The slightly lower overall prevalence of benzodiazepine use in our cohort compared with a recent surgical population likely reflects the inclusion of both medical and surgical patients.^11^ These findings are consistent with prior inpatient studies among older adults, which have reported frequent prescribing of benzodiazepines and Z-drugs in surgical departments, and an increased likelihood of potentially inappropriate psychotropic prescribing in this setting.^18^

Women were over-represented in both benzodiazepine groups, consistent with previous studies reporting higher benzodiazepine use among women,^11 19^ potentially related to a higher prevalence of anxiety and sleep-related complaints.^20^,^22^

Overall, these findings highlight missed opportunities for structured medication review, particularly for patients with chronic benzodiazepine use.^8^ At the same time, the feasibility of deprescribing during hospitalisation likely depends on the pattern and intensity of benzodiazepine use. While discontinuation may be feasible for patients using benzodiazepines on a PRN (as needed) basis, meaningful dose reduction or cessation among patients with daily or long-term use may be challenging during typically short hospital stays, particularly due to concerns about withdrawal symptoms. In these patients, hospitalisation may primarily offer an opportunity for reassessment, documentation and planning of deprescribing after discharge.

### Future research

These findings underscore the need for structured inpatient benzodiazepine management protocols, especially for patients already on these medications before admission. Such protocols could include a systematic review of indication, duration and appropriateness, with clear guidance for tapering or discontinuation when clinically suitable.

Although most newly initiated benzodiazepines were discontinued at discharge, a meaningful minority of patients were discharged with a new prescription. Future research could explore the factors associated with this continued use, and whether these prescriptions reflect appropriate indications or signal a risk of unintended long-term use.

### Strengths and limitations

A key strength of this study is the clear distinction made between patients with prior benzodiazepine use and those newly initiated during hospitalisation. This differentiation allows for a more nuanced understanding of prescribing behaviours and patient trajectories, highlighting how clinical decisions may differ based on a patient’s medication history. By examining medication status at both admission and discharge, the study captures critical transition points in care where the risk of inappropriate initiation or continuation is high. These transition moments are relevant for improving medication safety, as they offer important opportunities for reassessment, deprescribing and alignment with best practice guidelines.

This study has some limitations. Although we used real-world data extracted from electronic patient records, the dataset did not include detailed information on the duration of benzodiazepine use prior to admission or following discharge. This restricts conclusions about long-term prescribing patterns. Data from the WEsleep trial, which was an interventional study, were used for this analysis. Although the intervention may have introduced some awareness regarding benzodiazepine prescribing, no significant difference in use of benzodiazepines was observed between the intervention and control groups in the main study, suggesting a limited effect overall.^14^ However, the fact that this subanalysis was conducted in the context of an intervention study may still imply that prescribing behaviour was slightly more cautious than in usual care. As such, the limited review of ongoing benzodiazepine use observed here could actually underestimate how little attention is paid to this issue in routine clinical practice.

## Conclusion

This study identifies two notable findings in inpatient benzodiazepine prescribing. First, benzodiazepines initiated during hospitalisation were most often intended for short-term use, as the vast majority of newly started prescriptions were discontinued at discharge. Second, for patients who were already using benzodiazepines prior to admission, medication regimens were rarely reassessed or adjusted during hospitalisation. Despite overall cautious prescribing practices, nearly 1 in 10 patients without prior use were still discharged with a new benzodiazepine prescription, highlighting a risk of unintended continuation beyond the hospital stay. These findings suggest that while hospitals are taking steps in the right direction, there is still considerable room for improvement, both in preventing unnecessary continuation after short-term use and in re-evaluating chronic benzodiazepine therapy during admission.

To promote safe and rational use, hospitals should implement structured protocols for medication review, deprescribing and discharge planning, with a particular focus on non-pharmacological alternatives for common indications such as sleep disturbance. Our findings also support a more cautious and guideline-adherent prescribing approach by both hospital-based and community physicians. Strengthened collaboration with primary care is also essential to support long-term management and appropriate (de)prescribing beyond discharge.

## Supplementary material

10.1136/bmjopen-2025-112758 online supplemental file 1

10.1136/bmjopen-2025-112758 online supplemental file 2

## Data Availability

Data are available upon reasonable request.
