# Supplementary material for: Benzodiazepine receptor agonists in hospitalised patients in the Netherlands: initiation, continuation and discontinuation – a retrospective observational analysis
Source: BMJ Open. 2026 Feb 9;16(2):e112758. doi: 10.1136/bmjopen-2025-112758 (PMC12887526; doi:10.1136/bmjopen-2025-112758)
Supplement: online supplemental file 1 [file bmjopen-16-2-s001.docx]

**Supplementary material**

Table of contents

[Table 1. Prescribed benzodiazepines at home multiple times a day or during the daytime 2](#_Toc219805181)

[Table 2. Prescribed benzodiazepines at home for the evening or before bedtime 2](#_Toc219805182)

[Table 3. Benzodiazepines prescribed during hospital admission (daytime) 2](#_Toc219805183)

[Table 4. Benzodiazepines prescribed during hospital admission (afternoon/evening) 3](#_Toc219805184)

# Table 1. Prescribed benzodiazepines at home multiple times a day or during the daytime

|  | *n^a^* |
| --- | --- |
| Oxazepam 5-10-20-25mg | 24 |
| Lorazepam 0,5-1mg | 6 |
| Diazepam 5-10mg | 3 |
| Temazepam 10-20mg | 2 |
| Alprazolam 0,5mg | 1 |
| Clonazepam 0,5mg | 1 |
| Clorazepate 5mg | 1 |
| Lormetazepam 0,5mg | 1 |
| Midazolam 2,5-5mg | 1 |

*^a^* *^Frequencies may exceed the number of patients due to concomitant use of multiple benzodiazepines by individual patients.^*

# Table 2. Prescribed benzodiazepines at home for the evening or before bedtime

| *Name of benzodiazepine* | *n^b^* |
| --- | --- |
| Temazepam 10-20mg | 29 |
| Zolpidem 5-10mg | 9 |
| Zopiclone 3,75-7,5-15mg | 7 |
| Lormetazepam 0,5-1-2mg | 3 |
| Lorazepam 1-2,5mg | 3 |
| Diazepam 5-10mg | 2 |
| Oxazepam 5-10mg | 2 |
| Bromazepam 3mg | 1 |
| Clobazam 10mg | 1 |
| Clonazepam 2mg | 1 |
| Clorazepater 5mg | 1 |
| Midazolam 15mg | 1 |
| Nitrazepam 5mg | 1 |

*^b^* *^Frequencies may exceed the number of patients due to concomitant use of multiple benzodiazepines by individual patients.^*

# Table 3. Benzodiazepines prescribed during hospital admission (daytime)

|  | N^c^ |
| --- | --- |
| Oxazepam 10mg | 31 |
| Midazolam 7,5mg | 7 |
| Midazolam iv 1mg | 1 |
| Diazepam 10mg | 1 |
| Lorazepam 1mg | 1 |

*^c^* *^Frequencies may exceed the number of patients due to concomitant use of multiple benzodiazepines by individual patients.^*

# Table 4. Benzodiazepines prescribed during hospital admission (afternoon/evening)

|  | n^d^ |
| --- | --- |
| Temazepam 10mg | 52 |
| Oxazepam 5-10mg | 30 |
| Lorazepam 0,5-1-2mg | 7 |
| Zolpidem 5-10mg | 3 |
| Zopiclone 7,5mg | 3 |
| Midazolam 7,5mg | 3 |
| Diazepam 2-10mg | 2 |

*^d Frequencies may exceed the number of patients due to concomitant use of multiple benzodiazepines by individual patients.^*
