# Supplementary material for: Benzodiazepine receptor agonists in hospitalised patients in the Netherlands: initiation, continuation and discontinuation – a retrospective observational analysis
Source: BMJ Open. 2026 Feb 9;16(2):e112758. doi: 10.1136/bmjopen-2025-112758 (PMC12887526; doi:10.1136/bmjopen-2025-112758)
Supplement: online supplemental file 2 [file bmjopen-16-2-s002.docx]

**Supplement 2**

**The WEsleep trial**

Improving sleep quality in surgical and medical patients – A Randomized Clinical Trial

**PROTOCOL TITLE**

**(full): Improving sleep quality and circadian rhythm in surgical and medical patients using low-cost interventions**

| **Protocol ID** | **The WEsleep trial** |
| --- | --- |
| **Short title** | **Improving sleep quality in surgical and medical patients** |
| **METC number** | **W22_358** |
| **EudraCT number** | **NA** |
| **Registration number** | **NCT05683483**  **Clinicaltrials.gov**  https://clinicaltrials.gov/study/NCT05683483?cond=wesleep&rank=1 |
| **Version** | **1.0** |
| **Date** | ***05-01-2023*** |
| **Principal investigator** | **XXXXXXXX** |
| **Coordinating investigators:** | XXXXXXXXXXX |
| **Sponsor**  *Representative:* | **XXXXXXXXX** |
| **Subsidising party** | **Amsterdam University Medical Centers** |
| **Independent expert (s)** | **XXXXXXXX** |
| **Pharmacy** | **NA** |

Table of contents

[1. LIST OF ABBREVIATIONS AND RELEVANT DEFINITIONS 6](#_Toc178934174)

[2. SUMMARY 7](#_Toc178934175)

[3. INTRODUCTION AND RATIONAL 8](#_Toc178934176)

[3.1 Hypotheses 8](#_Toc178934177)

[OBJECTIVES 9](#_Toc178934178)

[3.2 Primary Objective: 9](#_Toc178934179)

[3.3 Secondary Objectives: 9](#_Toc178934180)

[4. STUDY DESIGN 11](#_Toc178934181)

[STUDY POPULATION 13](#_Toc178934182)

[4.1 Population (base) 13](#_Toc178934183)

[4.2 Inclusion criteria 13](#_Toc178934184)

[4.3 Exclusion criteria 13](#_Toc178934185)

[4.4 Sample size calculation 14](#_Toc178934186)

[5. TREATMENT OF SUBJECTS 15](#_Toc178934187)

[5.1 Investigational product/treatment 15](#_Toc178934188)

[5.2 Randomization 16](#_Toc178934189)

[6. INVESTIGATIONAL PRODUCT 17](#_Toc178934190)

[7. NON-INVESTIGATIONAL PRODUCT 17](#_Toc178934191)

[8. METHODS 18](#_Toc178934192)

[8.1 Study parameters/endpoints 18](#_Toc178934193)

[8.1.1 Main endpoint 18](#_Toc178934194)

[8.1.2 Time of main endpoint 18](#_Toc178934195)

[8.1.3 Secondary endpoints 18](#_Toc178934196)

[8.1.4 Time of other study parameters 19](#_Toc178934197)

[8.2 Randomisation, blinding and treatment allocation 19](#_Toc178934198)

[8.2.1 Screening and inclusion 20](#_Toc178934199)

[8.2.2 Randomization 20](#_Toc178934200)

[8.3 Withdrawal of individual subjects 20](#_Toc178934201)

[8.3.1 Specific criteria for withdrawal (if applicable) 20](#_Toc178934202)

[8.4 Replacement of individual subjects after withdrawal 20](#_Toc178934203)

[8.5 Follow-up of subjects withdrawn from treatment 20](#_Toc178934204)

[8.6 Premature termination of the study 20](#_Toc178934205)

[8.7 In case of postponement of surgery 20](#_Toc178934206)

[9. SAFETY REPORTING 21](#_Toc178934207)

[10. STATISTICAL ANALYSIS 21](#_Toc178934208)

[10.1 Primary study parameter 21](#_Toc178934209)

[10.1.1 Sample size calculation (cfr 4.4 Sample size) 21](#_Toc178934210)

[10.2 Secondary study parameter(s) 22](#_Toc178934211)

[11. ETHICAL CONSIDERATIONS 24](#_Toc178934212)

[11.1 Regulation statement 24](#_Toc178934213)

[11.2 Recruitment and consent 24](#_Toc178934214)

[11.3 Objection by minors or incapacitated subjects 24](#_Toc178934215)

[11.4 Benefits and risks assessment, group relatedness 24](#_Toc178934216)

[11.5 Compensation for injury 24](#_Toc178934217)

[12. ADMINISTRATIVE ASPECTS, MONITORING AND PUBLICATION 25](#_Toc178934218)

[12.1 Handling and storage of data and documents 25](#_Toc178934219)

[12.2 Monitoring and Quality Assurance 25](#_Toc178934220)

[12.3 Amendments 25](#_Toc178934221)

[12.4 Annual progress report 25](#_Toc178934222)

[12.5 Temporary halt and (prematurely) end of study report 25](#_Toc178934223)

[12.6 Public disclosure and publication policy 26](#_Toc178934224)

[13. STRUCTURED RISK ANALYSIS 26](#_Toc178934225)

[14. REFERENCES 27](#_Toc178934226)

# LIST OF ABBREVIATIONS AND RELEVANT DEFINITIONS

| **ABR** | **General Assessment and Registration form (ABR form), the application form that is required for submission to the accredited Ethics Committee; in Dutch: Algemeen Beoordelings- en Registratieformulier (ABR-formulier)** |
| --- | --- |
| **Sponsor** | **The sponsor is the party that commissions the organisation or performance of the research, for example a pharmaceutical**  **company, academic hospital, scientific organisation or investigator. A party that provides funding for a study but does not commission it is not regarded as the sponsor, but referred to as a subsidising party.** |
| **SUSAR** | **Suspected Unexpected Serious Adverse Reaction** |
| **UAVG** | **Dutch Act on Implementation of the General Data Protection Regulation; in Dutch: Uitvoeringswet AVG** |
| **WMO** | **Medical Research Involving Human Subjects Act; in Dutch: Wet Medisch-wetenschappelijk Onderzoek met Mensen** |

# SUMMARY

**Rationale:** The daily sleep-wake rhythm is strongly disturbed in hospitalized patients. There is a lot of research on easily applicable and low-cost interventions to improve sleep. However, many of these studies have inadequate sample sizes or suboptimal study designs.

**Objective**: To evaluate the effect of implementing easy applicable and low-cost

interventions to improve sleep quality in hospitalized surgical and medical patients.

**Study design:** Cluster Randomized Controlled trial

**Study population:** Surgical and medical hospitalized patients

**Intervention**: A combination of interventions, including noise and sleep disturbance reduction, education of health-care professionals on the importance of good sleep, postponing morning check-ups for vital signs when possible, optimizing medication and iv fluid timing, reducing after-hours food intake and a “sleep menu” with items patients can choose from to improve their sleep, such as earplugs and eye-masks.

**Main study endpoint:** Sleep quality measured by the Richards-campbells sleep questionnaire.

**Nature and extent of the burden and risks associated with participation, benefit and group relatedness:** Benefits include possible improvement in sleep quality on departments where the intervention is implemented. Burden of participation is filling out a baseline questionnaire at inclusion and a short sleep diary daily for every day of hospital admission. All interventions are very low-risk.

**Key words:** sleep, hospital, multicomponent intervention

# INTRODUCTION AND RATIONAL

Our body physiology is highly intertwined with our internal human clock or circadian timing system. The human circadian timing system consists of the central clock in the hypothalamic suprachiasmatic nucleus and peripheral clocks in other brain regions and all peripheral tissues. The circadian timing system regulates circadian timing systems (i.e., rhythms of ± 24 hours) in physiology including sleep-wake behavior and attention, the secretion of various hormones such as glucocorticoids and melatonin, and the activity of the autonomic nervous system. The driving mechanism of the various clocks in the circadian timing system is the transcriptional translational feedback loop (TTFL), consisting of core clock genes with several accessory feedback loops(1-6). To synchronize the various clocks to the 24 hour rhythm of the environment, clocks respond to “Zeitgebers”, i.e. external stimuli that affect the rhythm of the molecular clock. Disruption of the circadian timing system has been shown to have a broad negative impact, including on metabolism, the immune system, mood and cognitive functioning(7, 8). We have previously shown that hospital admission and surgery disrupt the circadian timing system and reduces sleep quality(9), thereby possibly disturbing the normal homeostasis and potentially increasing the risk of morbidity and mortality. Interventions to minimize circadian disruption and improve sleep quality are therefore needed. Evidence on different easily applicable interventions to improve sleep is increasing, although well designed randomized controlled trials in the hospital setting are lacking. Interventions are usually aimed at promoting activity and reducing disturbance of the circadian timing system during the day and improving sleep at night, usually by reducing sleep disturbing factors. Interventions that are proven to be effective are eye masks(10, 11), ear plugs(12), increasing daytime- and reducing nighttime light exposure(13), nighttime noise reduction (e.g. quiet time or intervention with posters to remind nurses during rounds)(14, 15), and education of nurses on the importance of sleep(16). Several other previous studies have shown multicomponent interventions to be effective as well(17-22). However, most previous studies investigated only a single intervention at the time. All but one of the studies that investigated a multicomponent set of interventions were not set up as a randomized controlled trial. Lastly, one RCT with a multicomponent intervention was performed(21), but the set of interventions was quite limited (only eye masks, ear plugs and a white noise machine with instructions on use and encouragement to do so).

The aim of this project is to study the effect of a comprehensive set of low-cost easy applicable non-pharmacological interventions on sleep quality in hospitalized surgical and medical patients.

## Hypotheses

We hypothesize that using easy applicable low-cost interventions, we will be able to minimize disruption of the circadian timing system and improve sleep quality in hospitalized surgical and medical patients.

## OBJECTIVES

## Primary Objective:

To investigate whether a comprehensive set of low-cost and easily applicable interventions improves sleep quality in hospitalized surgical and medical patients as measured by the subjective Richard Campbell Sleep Questionnaire on the second night of admission for medical patients and first night postoperatively for surgical patients.

## Secondary Objectives:

- To investigate whether the WEsleep interventions improves subjective sleep quality for all nights of admission (max. 7)
- To investigate whether the WEsleep interventions reduced the lowering of subjective sleep quality during admission when compared to subjective sleep quality before admission (on a work-free day 30 days before admission, retrospectively assessed at inclusion)
- To investigate whether the disruption of the circadian timing system (as measured by the diurnal phase shift of midpoint of sleep) was smaller for patients in the intervention group on the first postoperative night for surgical patients and the second night of admission for medical patients
- To investigate whether the WEsleep interventions reduced night-to-night changes in subjective sleep-wake timing (i.e. midpoint of sleep, on- and offset, duration, latency, inertia and daytime napping) for all nights of admission (max. 7)
- To investigate the association between the timing of food intake (i.e. first and last meal of the day and whether food was eaten after 21:00h) and sleep quality and timing.
- For Surgical patients: To investigate the association between quality of recovery and sleep quality and sleep-wake timing
- To investigate 30-day mortality for patients admitted into intervention and control departments
- To investigate 30-day incidence of delirium for patients admitted into intervention and control departments
- For surgical patients: to investigate the 30-day incidence of surgical site infections

Objective sleep substudy

- Objective sleep substudy: to investigate the difference between intervention and control departments in objective sleep efficiency of hospitalized patients on the second night of admission (in medical patients) and the first postoperative night on ward (in surgical patients) as measured by the Dreem-3 EEG-headband
- Objective sleep substudy: to investigate whether the WEsleep interventions reduced night-to-night changes in objective sleep-wake timing (i.e. midpoint of sleep, on- and offset, duration, latency, inertia and daytime napping) for all nights of admission (max. 7)
- Objective sleep substudy: to investigate whether the WEsleep interventions influence night-to-night changes in objective sleep measures (as measured by the Dreem 3 EEG-headband and Withings Sleep analyzer) for all nights of admission (max. 7)
- Objective sleep substudy: to investigate the difference between objective sleep measures (as measured by Dreem-3 EEG-headband and Withings Sleep Analyzer) and subjective sleep measures (as measured by the Richard Campbell Sleep Questionnaire and the Consensus Sleep Diary) for a population of hospitalized medical and surgical patients
- Objective sleep substudy: to investigate the association between quality of recovery and objective sleep quality and sleep-wake timing
- Objective sleep substudy: to investigate the association between 30-day mortality and objective sleep quality and quantity
- Objective sleep substudy: to investigate the association between 30-day incidence of delirium and objective sleep quality and quantity
- Objective sleep substudy: to investigate the association between the 30-day incidence of surgical site infections and objective sleep quality and quantity (for surgical patients)

# STUDY DESIGN

In this cluster randomized controlled trial, performed in both locations of the Amsterdam University Medical Centre, we will randomize 6 surgical and 6 medical departments to either standard care group (SG) or WEsleep intervention group (WI). All adult patients admitted to the participating departments will receive the sleep improving interventions during their admission, informed consent will be obtained for sleep assessment.

Implementation of the interventions will be evaluated on a regular basis throughout the length of the study to make sure that all interventions are provided to our patients as planned. Before implementation, a baseline measurement will be conducted on all participating departments in a small number of patients (which is not part of the sample size). We observed that some interventions were gradually implemented. As such, we decided that for optimal assessment of the effect of the intervention a run-in period of 12 weeks before starting assessment of the primary and secondary outcomes would be reasonable. Sleep measurements between study onset and outcome will be used to guide implementation and will be reported in eventual journal articles.

After 12 weeks (that is, after July 1st), 33 patients per participating department will be included, for a total of 396 patients (12 departments with 33 patients each) and analyzed for the outcomes of the study. Informed consent for sleep measurements will be obtained on the first or second day of admission into one of the participating wards. Patients will be followed until discharge or for a maximum of 7 nights of admission. Patient characteristics, as well as data on the admission will be extracted from the electronic medical record. Patients will be asked to complete two questionnaires daily: Richards Campbell Sleep Questionnaire (RCSQ) on subjective sleep quality and the Consensus Sleep Diary (CSD) for day-to-day sleep wake timing. Furthermore, patients are asked at admission to complete the two questionnaires on sleep quality and sleep-wake timing on work- and work-free days (RCSQ and CSD) retrospectively, i.e. for 30 days before admission. Surgical patients will also complete a Quality of Recovery 15 (QoR-15) questionnaire twice (once at one day after surgery/first day of admission and once three days after surgery/third day of admission).

In the separate objective sleep substudy, 24 patients will be recruited from each group (medical intervention and control groups, surgical intervention and control groups), for a total of 96 patients. Measurements between April 11th and June 30th will be used to guide implementation of interventions. Patients included after July 1st will be analyzed for the outcomes of the substudy. Informed consent for the substudy will be obtained separately at the same time as consent for the main study. All patients participating in the main study will be asked to participate in the substudy as well. After obtaining consent, a lottery system will decide which patients can participate in the substudy (due to the scarcity of available Dreem-3 EEG headbands and Withings Sleep Analyzers).

In the substudy, objective sleep in patients will be measured with the Dreem-3 EEG-headband and the Withings Sleep Analyzer. The Dreem-3 headband uses electro-encephalography (EEG) and a validated algorithm to differentiate between wakefulness and various sleep stages (Rapid-Eye Movement (REM)-sleep and non-REM N1, N2 and N3 sleep) and the time at which these occur. The Withings Sleep Analyzer can be placed under the mattress and uses measurement of movement to differentiate between wakefulness and light, deep and REM-sleep and the times at which these occur.

# STUDY POPULATION

## Population (base)

A) Surgical patients: Patients admitted for elective surgery with planned overnight stay

B) Medical patients: Patients admitted to the medical ward, expected to stay for at least two nights.

As randomization will take place at the department levels (as clusters) rather than at the individual patient level, all patients that are admitted will receive the interventions, but data will be collected only in patients that meet the following inclusion criteria.

## Inclusion criteria

In order to be eligible to participate in data collection for this study, a subject must meet all of the following criteria:

A) (elective surgery patients)

- Adult (18+) patients
- Undergoing elective non-cardiac surgery with planned (postoperative) overnight stay
- Willing and able to provide informed consent
- Able to read and write in Dutch

B) (medical patients)

- Adult (18+) patients on medical departments with expected stay of at least two nights.
- Willing and able to provide informed consent
- Able to read and write in Dutch

## Exclusion criteria

A potential subject who meets any of the following criteria will be excluded from participation in this study:

- Surgical patients: ASA score of 4 or higher
- Surgical patients: PACU admission during first postoperative night
- Planned admission on ICU-ward at any time during admission (for both surgical and medical patients)
- Non-Dutch speaking
- Strict isolation (MRSA, aerogenic)
- Pre-existing delirium or cognitive impairment at inclusion

NB: After starting inclusions, some patients were found to spend the first postoperative night on post-anesthesia care unit (PACU) instead of a WEsleep intervention or control department and after deliberation this was added as a criterium for exclusion.

## Sample size calculation

**Sample size for measuring difference in sleep quality measured by RCSQ between intervention and control group**

Sample sizes of 99 in group SG and 99 in group WG which were obtained by sampling 3 clusters with an average of 33 subjects each in group one and 3 clusters with an average of 33 subjects each in group two, achieve 80% power to detect a difference on the RCSQ between the group means of at least 7.7. We have obtained this difference from Unal et al, using the RCSQ to assess the relation between sleep and delirium prevalence(23). The standard deviation of subjects is 10.27. The intracluster correlation coefficient (ICC) is difficult to predict so we selected a conservative ICC of 0.030. The coefficient of variation of cluster sizes is 0.000. A two-sided t-test was used with a significance level of 0.050. This test used degrees of freedom based on the number of clusters.

In conclusion:

Medical patients:

Intervention (WG): 99

Control group (SG): 99

Surgical patients:

Intervention (WG): 99

Control group (SG): 99

Total: 396 patients.

**Sample size EEG headband**

We chose sleep efficiency as our primary outcome on the substudy with EEG-headbands. There are no studies using either the Philips or Dreem Headband to compare sleep in two groups. However, polysomnography (PSG) also uses an EEG signal. One study studying sleep parameters using PSG compared groups before either a low or high dose of melatonin, and found a statistical significant difference of 6.24 with a standard error of 1.81 (24). This yields a sample size of 3 in each group, using an unpaired t-test with a power of 80% and significance level of 0.05.

# TREATMENT OF SUBJECTS

## Investigational product/treatment

We will randomize departments to the Wesleep group (WI) and the standard care group (SC).

*WEsleep interventions*

All interventions will be divided into four categories:

- Category 1: Morning rounds of the nurses:
  - If possible, morning rounds for vital signs will be postponed from night shift (5:30-7:30 am) to the day shift (7:30-8:45 am).
  - Nurses will ask patients about their sleep quality, using a numeric rating scale. The results will be discussed during the morning rounds with physicians.
- Category 2: At admission and during morning rounds
  - A patient information brochure on sleep hygiene will be provided to all patients. This will have information about the importance of sleep and sleep hygiene and ‘tips and tricks’ to optimize sleep. It will also include a sleep menu (see category 4).
  - A smartphrase (short morning round checklist) in the electronic patient record (Epic) will be provided to physicians/nurses of participating departments. Physicians and nurses will be encouraged to use this during rounds to monitor patient sleep.
  - Intravenous fluids will be discontinued in the late evening and night, provided it is medically safe and responsible to do so.
  - If patients take corticosteroids or diuretics, these will be stimulated to be given in the morning, provided it is medically safe and responsible to do so. This is because administering corticosteroids in the morning mimics the endogenous cortisol diurnal cycle, while it has also been proven that corticosteroids in the evening negatively impacts sleep. For diuretics, evening administration prevents nighttime awakening for urinating.
- Category 3: Education of health care professionals and department infrastructure
  - Several clinical training sessions will be provided on the importance of sleep and sleep hygiene for both nurses and physicians.
  - Informative posters to remind staff about sleep and sleep hygiene will be put on all participating wards.
  - Noise and light exposure (e.g. alarms from infusion pumps and unnecessary nighttime hallway lighting), will be reduced to a minimum during the night.
  - Implement blackout curtains depending on baseline light intensity
- Category 4: Implementing sleep rounds
  - Sleep rounds will be implemented. During sleep rounds, patients will be reminded to visit the bathroom before bedtime, and IV bags will be switched to prevent having to change them while the patient sleeps.
  - A sleep menu will be provided to the patients with the patient information brochure. This will include items patients might use to improve their sleep, i.e. a sleep mask, ear plugs and warm blankets and socks.
  - Late night snacking (after 21.00 hours) will be discouraged wherever possible

In the control group care as usual is provided according to the current protocols.

## Randomization

As this is a cluster randomized controlled trial, patients will not be randomized individually. 12 departments will be asked to participate in the trial (6 medical and 6 surgical departments. Medical and surgical departments will be randomized separately, using a standard method of randomization (e.g. randomization.com). This will yield 3 medical en 3 surgical departments in both groups (intervention vs. control). The Intensive Care Unit and the surgical department for Cardiothoracic Surgery will be excluded from randomization. Inclusion of departments will stop when 6 medical and 6 surgical departments are found that are willing to participate.
3 medical and 3 surgical departments will provide care using the WEsleep-protocol from the start of the trial. The other departments will provide standard care during this time.

After the study, we will also implement the interventions at the control departments, as long as the preliminary results are positive.

# INVESTIGATIONAL PRODUCT

**Only applicable for the subjective sleepstudy.**

**Dreem headband**

The Dreem Headband is a compact system for recording brain activity (EEG), movements and breathing frequency

The Dreem can measure: sleep endpoints and quality metrics (determined following the American Academy of Sleep Medicine Definition), hypnograms with associated sleep stages, raw data files, which include EEG channels and breathing rate.

The Dreem 3 headband for research is registered as an FDA Class II Medical Device in the USA.
The Dreem 3 headband is CE marked for electromagnetic compatibility but not considered as a medical device in Europe. It can be used as an investigational device in research studies

Dreem subjects' accounts will be created with pseudonymized names, ages, and gender for the study. Only the main investigator or authorized personnel will have access to the correspondence table that contains both the identity of the subjects and identifiers. Dreem does not have access to any personal data. Upon request, data can be deleted at any time during the study. At the end of the study (or after a few months depending on the agreement), Dreem will delete all the recordings from its server (25).

**Philips Smartsleep Deep Sleep headband**

The Philips Smartsleep Deep Sleep Headband is a wearable system for recording brain acitivity. It measures all sleep metrics.

Measurements are saved offline on the device itself, so only authorized personnel will have access to the results.

It is not a medical device and can be freely bought by people in Europe. It is CE marked (26).

**Withings Sleep Analyzer**

The Withings Sleep analyzer is a system which is placed under a person’s mattress, tracking heart and breathing rates and movement. By monitoring these parameters it is able to differentiate between REM and light and deep non-REM sleep, while also being able to detect sleep apnea disorders.

The Withings Sleep Analyzer can be freely bought by people in Europe and is CE-marked. It has also been used extensively in medical research to track sleep(27).

# NON-INVESTIGATIONAL PRODUCT

Not applicable.

# METHODS

## Study parameters/endpoints

### Main endpoint

Our primary outcome is the subjective sleep quality as measured the Richards-campbells sleep questionnaire.

Subjective sleep quality will be assessed with the Richard Campbell Sleep Questionnaire (RCSQ). The RCSQ measures sleep quality on five questions using a visual analogue scale (VAS), ranging from 0 to 100, with higher scores indicating a better sleep quality. A sixth question was added to assess noise levels (as is quite customary), using a VAS-scale, ranging from 0-100, with a higher score indicating more noise.

### Time of main endpoint

Main endpoint will be measured on the second night of admission for medical patients, first night after surgery for surgical patients.

### Secondary endpoints

- Night-to night changes in subjective sleep quality as measured with the RCSQ.
- Difference in subjective sleep quality between during admission and at home. For this outcome, we will assess the subjective sleep quality at home retrospectively at inclusion.
- Diurnal phase shift of sleep-wake timing: The phase shift of sleep-wake timing will be assessed by comparing midpoint of sleep during admission with midpoint of sleep on a work-free day before admission, both measured with the Consensus Sleep Diary (CSD). For this outcome, we will assess sleep-wake timing at home retrospectively at inclusion.
- Night-to-night changes in sleep-wake timing: The changes in sleep-wake timing (i.e. midpoint of sleep, sleep on- and offset time, sleep duration, sleep latency & inertia and daytime napping), as measured by the CSD.
- Timing of food intake and sleep quality/timing: the association between food intake timing (i.e. first and last meal of the day and whether food was eaten after 21:00h), as measured by asking patients directly for the time of the first and last meal of the day and whether patients ate after 21:00h), and subjective sleep quality and sleep-wake timing (as measured by the Richard Campbell Sleep Questionnaire
- Quality of recovery (for surgical patients) and sleep quality/timing. The association between quality of recovery (in surgical patients), as measured by the Quality-of-Recovery 15 item questionnaire (QoR-15), and subjective sleep quality and sleep-wake timing (as measured by the Richard Campbell Sleep Questionnaire and Consensus Sleep Diary respectively). The QoR-15 will be completed twice by patients.
- 30 day mortality, assessed by checking the medical record retrospectively 30 days after admission.
- 30-day incidence of delirium during admission, as assessed by a physician during admission. Patients will be assessed for delirium at the request of nursing staff or if the score of the Delirium Observation Screening scale is 3 or higher. The Delirium Observation Screening scale ranges from 0 to 39, with higher scores indicating a higher chance of delirium.
- 30-day incidence of surgical site infections (for surgical patients), as assessed by physicians from the department where patient is admitted during hospitalization. If the patient obtained a surgical site infection after discharge, incidence was assessed in the outpatient clinic by a physician from the department where patient was admitted.

Objective sleep substudy:

- Sleep efficiency (objectively measured) during admission. Objective sleep quality will be measured by the Dreem-3 headband, using electro-encephalography (EEG) to differentiate between wakefulness, Rapid-Eye-Movement (REM) sleep and non-REM N1, N2 and N3 sleep. The primary outcome measure will be sleep efficiency (time slept divided by time in bed spent trying to sleep), compared for patients on intervention and control departments.
- Differences in objective sleep measures of sleep-wake timing during admission. Changes in sleep-wake timing (i.e. midpoint of sleep, sleep on- and offset time, sleep duration, sleep latency & inertia) will be compared for patients on intervention and control departments.
- Night-to-night changes in objective sleep measures during admission. The amount of time in each of the different sleep stages and their distribution, as well as total sleep time, will be compared for patients on intervention and control departments.
- Difference between objective and subjective sleep measures during admission. Subjective sleep measured with the RCSQ and CSD.
- Quality of recovery after surgery and objective sleep quality/timing.
- Correlation 30 day mortality and objective sleep quality/timing.
- Correlation 30-day incidence of delirium during admission and objective sleep quality/timing.
- Correlation 30-day incidence of surgical site infections (for surgical patients) and objective sleep quality/timing.

### Time of other study parameters

Secondary endpoints will be measured during admission with a maximum of seven days. If a patient leaves the hospital before these seven days, the measurements will be stopped.

## Randomisation, blinding and treatment allocation

After inclusion in the trial, departments will be randomised by an investigator through online software (e.g. randomization.com) and according to Good Clinical Practice guidelines.

All patients admitted into one of the randomized departments is eligible to participate in the study, provided that the inclusion criteria are met.

**Study procedures**

After obtaining informed consent, patients are provided with the RCSQ and CSD for each day of admission, as well as for a regular night at home (one workday and one work-free day). Filled out questionnaires will be collected daily. Baseline characteristics and relevant data on admission (such as morbidity, length of hospital stay, etc.) will be collected from the patient records. Discharge from the hospital will be the end of the study for each participant.

### Screening and inclusion

Patients will be screened for eligibility by checking patient records of patients admitted into each of the participating departments. Patients that have objected to participating in medical studies will not be approached or screened for eligibility, to comply with AVG requirements.

Patients eligible will be approached at the department they are admitted at and will be included after informed consent has been obtained.

### Randomization

After completion of informed consent procedure, baseline characteristics will be noted. Randomization is on cluster/department level, not individual level.

## Withdrawal of individual subjects

Subjects can object to the use of their anonymized data at any time without any consequences. The investigator can decide to withdraw a subject from the study for urgent medical reasons.

### Specific criteria for withdrawal (if applicable)

Subjects may be removed from the study if any of the following events occur

- Refusal of the subject to continue observations
- Decision by the investigator or healthcare professional that termination is in the subject’s best medical interest

## Replacement of individual subjects after withdrawal

All drop-outs will be replaced

## Follow-up of subjects withdrawn from treatment

Not applicable.

## Premature termination of the study

As there are no risks associated with the study, premature termination and safety reporting is not included in this study protocol.

## In case of postponement of surgery

Not applicable.

# SAFETY REPORTING

As there are no risks associated with the study, premature termination and safety reporting is not included in this study protocol.

# STATISTICAL ANALYSIS

All patients with a total RCSQ score on the primary outcome night (second night of admission for medical patients and first night postoperatively for surgical patients) will be included in the final analysis. The baseline characteristics of all subjects, and per treatment group, will be outlined in a table describing variables such as demographic variables, weight, length, relevant medical history, current medication use, current ASA classification and type of anaesthesia. Continuous data will be presented as mean with standard deviation (SD) or as median with interquartile range (IQR), depending on the distribution of the data. Drop-outs will be replaced. Confounding will be evaluated using a linear regression analysis.

## Primary study parameter

Our primary study parameter is the subjective sleep quality measured by the RCSQ on the second night of admission for medical patients or the first night postoperatively for surgical patients. These were compared between patients admitted into intervention vs. control departments. If data are distributed normally, an unpaired two sided t-test with a significance level of 0.050 will be performed to compare the sleep quality of the intervention group with the control group. If data are not normally distributed, the equivalent non-parametric test will be used.

### Sample size calculation (cfr 4.4 Sample size)

**Sample size for measuring difference in sleep quality measured by RCSQ between intervention and control group**

Sample sizes of 99 in group SG and 99 in group WG which were obtained by sampling 3 clusters with an average of 33 subjects each in group one and 3 clusters with an average of 33 subjects each in group two, achieve 80% power to detect a difference on the RCSQ between the group means of at least 7.7. We have obtained this difference from a study using RCSQ to assess the relation between sleep and delirium prevalence(23). The standard deviation of subjects is 10.27. The intracluster correlation coefficient is difficult to predict so we have chosen to be on the safe side by choosing 0.030. The coefficient of variation of cluster sizes is 0.000. A two-sided t-test was used with a significance level of 0.050. This test used degrees of freedom based on the number of clusters.

In conclusion:

Medical patients:

Intervention (WG): 99

Control group (SG): 99

Surgical patients:

Intervention (WG): 99

Control group (SG): 99

Total: 396 patients.

**Sample size EEG headband**

We chose sleep efficiency as our primary outcome on the substudy with EEG-headbands. There are no studies using either the Philips or Dreem Headband to compare sleep in two groups. However, polysomnography (PSG) also uses an EEG signal. One study studying sleep parameters using PSG compared groups before either a low or high dose of melatonin, and found a statistical significant difference of 6.24 with a standard error of 1.81 (24). This yields a sample size of 3 in each group, using an unpaired t-test with a power of 80% and significance level of 0.05.

## Secondary study parameter(s)

- Night-to night changes in subjective sleep quality as measured with the RCSQ. To compare this a repeated measures ANOVA will be performed.
- Difference in subjective sleep quality between hospital and at home. For this outcome, we will assess the subjective sleep quality at home retrospectively at inclusion. To compare this a paired t-test with a significance level of 0.05 will be performed.
- Diurnal phase shift of sleep-wake timing: The phase shift of sleep-wake timing will be assessed by comparing midpoint of sleep during admission with midpoint of sleep on a work-free day before admission, both measured with the Consensus Sleep Diary (CSD). For this outcome, we will assess sleep-wake timing at home retrospectively at inclusion. To compare this a paired t-test with a significance level of 0.05 will be performed.
- Night-to-night changes in sleep-wake timing: The changes in sleep-wake timing (i.e. midpoint of sleep, sleep on- and offset time, sleep duration, sleep latency & inertia and daytime napping), as measured by the CSD. To compare this a repeated measures ANOVA will be performed.
- Timing of food intake and sleep quality/timing: the association between food intake timing (i.e. first and last meal of the day and whether food was eaten after 21:00h), as measured by asking patients directly for the time of the first and last meal of the day and whether patients ate after 21:00h), and subjective sleep quality and sleep-wake timing (as measured by the Richard Campbell Sleep Questionnaire. A linear regression will be performed for food timing as continuous variable, and a logistic regression will be performed for food timing after 21.00h as dichotomous variable.
- Quality of recovery (for surgical patients) and sleep quality/timing. The association between quality of recovery (in surgical patients), as measured by the Quality-of-Recovery 15 item questionnaire (QoR-15), and subjective sleep quality and sleep-wake timing (as measured by the Richard Campbell Sleep Questionnaire and Consensus Sleep Diary respectively). The QoR-15 will be completed twice by patients. A linear regression will be performed.
- 30 day mortality, assessed by checking the medical record retrospectively 30 days after admission. This will be compared between the intervention and control group by performing a Chi-square test.
- 30-day incidence of delirium during admission, as assessed by a physician during admission. Patients will be assessed for delirium at the request of nursing staff or if the score of the Delirium Observation Screening scale is 3 or higher. The Delirium Observation Screening scale ranges from 0 to 39, with higher scores indicating a higher chance of delirium. This incidence will be compared between the intervention and control group by performing a Chi-square test.
- 30-day incidence of surgical site infections (for surgical patients), as assessed by physicians from the department where patient is admitted during hospitalization. If the patient obtained a surgical site infection after discharge, incidence was assessed in the outpatient clinic by a physician from the department where patient was admitted. This will be compared between the intervention and control group by performing a Chi-square test.

Objective sleep substudy:

- Sleep efficiency (objectively measured) during admission. Objective sleep quality will be measured by the Dreem-3 headband, using electro-encephalography (EEG) to differentiate between wakefulness, Rapid-Eye-Movement (REM) sleep and non-REM N1, N2 and N3 sleep. The primary outcome measure will be sleep efficiency (time slept divided by time in bed spent trying to sleep), compared for patients on intervention and control departments. If data are distributed normally, an unpaired two sided t-test with a significance level of 0.050 will be performed to compare the sleep quality of the intervention group with the control group. If data are not normally distributed, the equivalent non-parametric test will be used.
- Differences in objective sleep measures of sleep-wake timing during admission. Changes in sleep-wake timing (i.e. midpoint of sleep, sleep on- and offset time, sleep duration, sleep latency & inertia) will be compared for patients on intervention and control departments. If data are distributed normally, an unpaired two sided t-test with a significance level of 0.050 will be performed to compare the sleep quality of the intervention group with the control group. If data are not normally distributed, the equivalent non-parametric test will be used.
- Night-to-night changes in objective sleep measures during admission. To compare this a repeated measures ANOVA will be performed.
- Difference between objective and subjective sleep measures during admission. Subjective sleep measured with the RCSQ and CSD. This will be evaluated by looking at the association between objective and subjective sleep by performing linear regression.
- Quality of recovery after surgery and objective sleep quality/timing. This will be evaluated by performing linear regression.
- Correlation 30 day mortality and objective sleep quality/timing. This will be evaluated by performing linear regression.
- Correlation 30-day incidence of delirium during admission and objective sleep quality/timing. This will be evaluated by performing linear regression.
- Correlation 30-day incidence of surgical site infections (for surgical patients) and objective sleep quality/timing. This will be evaluated by performing linear regression.

# ETHICAL CONSIDERATIONS

## Regulation statement

The study will be conducted according to the principles of the Declaration of Helsinki 2004 (amendment Fortaleza, Brazil, October 2013) and in accordance with the Medical Research Involving Human Subjects Act (WMO) and CCMO/ICH GCP guidelines.

## Recruitment and consent

Patients admitted into one of the participating departments who meet inclusion criteria will be asked if they are willing to participate in the evaluation of the intervention at the first day of hospital admission (or as soon as possible, if recruitment cannot take place on the first day (e.g. admission in the weekend)). Patients will be recruited after informed consent has been obtained.

## Objection by minors or incapacitated subjects

No minors or incapacitated subjects will be included in this study.

## Benefits and risks assessment, group relatedness

Patients admitted into the participating departments that are randomized for intervention will have the possible benefits of the interventions aimed at an improved sleep quality and minimal disruption of the circadian timing system. All interventions are very low-risk, as they are intended to be easily implemented and applied.

Patients admitted into control departments will receive standard care and will have no additional benefits or risks associated with the study.

## Compensation for injury

The sponsor/investigator has a liability insurance which is in accordance with article 7 of the WMO. The sponsor (also) has an insurance which is in accordance with the legal requirements in the Netherlands (Article 7 WMO). This insurance provides cover for damage to research subjects through injury or death caused by the study.

The insurance applies to the damage that becomes apparent during the study or within 4 years after the end of the study.

# ADMINISTRATIVE ASPECTS, MONITORING AND PUBLICATION

## Handling and storage of data and documents

The data of each patient will be noted on an individual case report form (electronic Case Report Form (CRF). All data of each patient will be noted on an individual case report form. Data will be coded using a numerical code, the key to this code is only available to the research team and is stored in the investigator site file in accordance with the Dutch law ‘Wet Bescherming Persoonsgegevens’ (WBP; Personal Data Protection Act) and GCP. All patient data will be handled confidentially and anonymously. Data will then be inserted into a database (GCP validated), and correctness of entries will be controlled by a second investigator. All data, including case report forms, trial master file, investigator site file and consent forms will be stored for 15 years after completion of the study. Data, both anonymous (e.g. CRFs) or not (e.g. source documents), will always be stored securely, in a locked cabinet (hard copy) or on password secured computers.

## Monitoring and Quality Assurance

Monitoring will be performed according to Dutch Federation of University Medical Centre norms, NFU 2.0). A detailed monitoring plan will be drafted, which involves an initiation visit and subsequent yearly monitoring visits for each site involved. Monitoring is performed by the Clinical Research Unit (CRU, according to Dutch Federation of University Medical Centre norms, NFU 2.0). In addition, the study can be subjected to internal and external auditing.

## Amendments

A ‘substantial amendment’ is defined as an amendment to the terms of the METC application, or to the protocol or any other supporting documentation, that is likely to affect to a significant degree:

- the safety or physical or mental integrity of the subjects of the trial;
- the scientific value of the trial;
- the conduct or management of the trial; or
- the quality or safety of any intervention used in the trial.

All substantial amendments will be notified to the METC and to the competent authority.

Non-substantial amendments will not be notified to the accredited METC and the competent authority, but will be recorded and filed by the sponsor.

## Annual progress report

The sponsor/investigator will submit a summary of the progress of the trial to the accredited METC once a year. Information will be provided on the date of inclusion of the first subject, numbers of subjects included and numbers of subjects that have completed the trial, serious adverse events/ serious adverse reactions, other problems, and amendments.

## Temporary halt and (prematurely) end of study report

The sponsor will notify the accredited METC and the competent authority of the end of the study within a period of 90 days. The end of the study is defined as the last patient’s last visit.

The sponsor will notify the METC immediately of a temporary halt of the study, including the reason of such an action.

In case the study is ended prematurely, the sponsor will notify the accredited METC and the competent authority within 15 days, including the reasons for the premature termination.
Within one year after the end of the study, the investigator/sponsor will submit a final study report with the results of the study, including any publications/abstracts of the study, to the accredited METC and the Competent Authority.

## Public disclosure and publication policy

There are no publication disclosures. The data will be presented on national and international congresses and will be published in a peer reviewed international journal.

# STRUCTURED RISK ANALYSIS

There are no risks associated with the study.

# REFERENCES

1. Reppert SM, Weaver DR. Coordination of circadian timing in mammals. Nature. 2002;418(6901):935-41.

2. Dibner C, Schibler U, Albrecht U. The mammalian circadian timing system: organization and coordination of central and peripheral clocks. Annu Rev Physiol. 2010;72:517-49.

3. Buckley TM, Schatzberg AF. On the interactions of the hypothalamic-pituitary-adrenal (HPA) axis and sleep: normal HPA axis activity and circadian rhythm, exemplary sleep disorders. J Clin Endocrinol Metab. 2005;90(5):3106-14.

4. Cipolla-Neto J, Amaral FGD. Melatonin as a Hormone: New Physiological and Clinical Insights. Endocr Rev. 2018;39(6):990-1028.

5. Claustrat B, Brun J, Chazot G. The basic physiology and pathophysiology of melatonin. Sleep Med Rev. 2005;9(1):11-24.

6. Morris CJ, Aeschbach D, Scheer FA. Circadian system, sleep and endocrinology. Mol Cell Endocrinol. 2012;349(1):91-104.

7. Stenvers DJ, Scheer F, Schrauwen P, la Fleur SE, Kalsbeek A. Circadian clocks and insulin resistance. Nat Rev Endocrinol. 2019;15(2):75-89.

8. Reid KJ, McGee-Koch LL, Zee PC. Cognition in circadian rhythm sleep disorders. Prog Brain Res. 2011;190:3-20.

9. van Zuylen ML, Meewisse AJG, Ten Hoope W, Eshuis WJ, Hollmann MW, Preckel B, et al. Effects of surgery and general anaesthesia on sleep-wake timing: CLOCKS observational study. Anaesthesia. 2022;77(1):73-81.

10. Babaii A, Adib-Hajbaghery M, Hajibagheri A. Effect of Using Eye Mask on Sleep Quality in Cardiac Patients: A Randomized Controlled Trial. Nurs Midwifery Stud. 2015;4(4):e28332.

11. Daneshmandi M, Neiseh F, SadeghiShermeh M, Ebadi A. Effect of eye mask on sleep quality in patients with acute coronary syndrome. J Caring Sci. 2012;1(3):135-43.

12. Sweity S, Finlay A, Lees C, Monk A, Sherpa T, Wade D. SleepSure: a pilot randomized-controlled trial to assess the effects of eye masks and earplugs on the quality of sleep for patients in hospital. Clin Rehabil. 2019;33(2):253-61.

13. Giménez MC, Geerdinck LM, Versteylen M, Leffers P, Meekes GJ, Herremans H, et al. Patient room lighting influences on sleep, appraisal and mood in hospitalized people. J Sleep Res. 2017;26(2):236-46.

14. Ho AGT, Tan M, Perez HM, Tan CN, Mordiffi SZ. Conducive environments reduce sleep disturbances and improve sleep quality: a quality improvement project. JBI Evid Implement. 2021;19(1):105-17.

15. Gardner G, Collins C, Osborne S, Henderson A, Eastwood M. Creating a therapeutic environment: a non-randomised controlled trial of a quiet time intervention for patients in acute care. Int J Nurs Stud. 2009;46(6):778-86.

16. Zhang P, Zhang Y, Han X, Zhang X, Zhu X, Li T. Effect of individualized psychological intervention on negative emotion and sleep quality of patients after bladder cancer surgery: a randomized controlled trial. Transl Androl Urol. 2021;10(7):3021-9.

17. Herscher M, Mikhaylov D, Barazani S, Sastow D, Yeo I, Dunn AS, et al. A Sleep Hygiene Intervention to Improve Sleep Quality for Hospitalized Patients. Jt Comm J Qual Patient Saf. 2021;47(6):343-6.

18. Gathecha E, Rios R, Buenaver LF, Landis R, Howell E, Wright S. Pilot study aiming to support sleep quality and duration during hospitalizations. J Hosp Med. 2016;11(7):467-72.

19. Lareau R, Benson L, Watcharotone K, Manguba G. Examining the feasibility of implementing specific nursing interventions to promote sleep in hospitalized elderly patients. Geriatr Nurs. 2008;29(3):197-206.

20. Antonio CK. Improving Quiet at Night on a Telemetry Unit: Introducing a Holistic Sleep Menu Intervention. Am J Nurs. 2020;120(10):58-64.

21. Farrehi PM, Clore KR, Scott JR, Vanini G, Clauw DJ. Efficacy of Sleep Tool Education During Hospitalization: A Randomized Controlled Trial. Am J Med. 2016;129(12):1329.e9-.e17.

22. Bartick MC, Thai X, Schmidt T, Altaye A, Solet JM. Decrease in as-needed sedative use by limiting nighttime sleep disruptions from hospital staff. J Hosp Med. 2010;5(3):E20-4.

23. Unal N, Guvenc G, Naharci M. Evaluation of the effectiveness of delirium prevention care protocol for the patients with hip fracture: A randomised controlled study. J Clin Nurs. 2022;31(7-8):1082-94.

24. Duffy JF, Wang W, Ronda JM, Czeisler CA. High dose melatonin increases sleep duration during nighttime and daytime sleep episodes in older adults. J Pineal Res. 2022;73(1):e12801.

25. Dreem. Brochure Dreem 3 for clinical research’. 2021.

26. Philips. Smartsleep Deep sleep headband [Available from: <https://www.usa.philips.com/c-e/smartsleep/deep-sleep-headband.html>.

27. Edouard P, Campo D, Bartet P, Yang RY, Bruyneel M, Roisman G, et al. Validation of the Withings Sleep Analyzer, an under-the-mattress device for the detection of moderate-severe sleep apnea syndrome. J Clin Sleep Med. 2021;17(6):1217-27.
