# Supplementary material for: Benzodiazepine receptor agonists in hospitalised patients in the Netherlands: initiation, continuation and discontinuation – a retrospective observational analysis
Source: BMJ Open. 2026 Feb 9;16(2):e112758. doi: 10.1136/bmjopen-2025-112758 (PMC12887526; doi:10.1136/bmjopen-2025-112758)
Supplement: Supplementary file 3 [file bmjopen-2025-112758.reviewer_comments.pdf]

PEER REVIEW HISTORY

BMJ Open publishes all reviews undertaken for accepted manuscripts. Reviewers are asked to complete a checklist review form and are provided with free text boxes to elaborate on their assessment. These free text comments are reproduced below.

ARTICLE DETAILS

Title (Provisional)

Benzodiazepine Receptor Agonists in Hospitalized Patients in the Netherlands: Initiation, Continuation, and Discontinuation – A retrospective observational analysis

Authors

De Gans, Carlijn J.; van den Ende, Eva S; Meewisse, Arjen J.G.; van Zuylen, Mark L.; Stenvers, Dirk Jan; Hermanides, Jeroen; Nanayakkara, Prabath W.

VERSION 1 - REVIEW

Reviewer 1

Name Himmel, Wolfgang

Affiliation University Medical Center Göttingen, Department of General Practice/Family Medicine

Date 12-Dec-2025

COI I'm co-author of two of the papers I recommend to be included in the manuscript (https://pubmed.ncbi.nlm.nih.gov/29202811/; https://pubmed.ncbi.nlm.nih.gov/31179613/).

The use of benzodiazepines in hospitals and in the community is still very high—despite warnings against their use, especially their long-term use. Any information about their prescribing patterns and possible reasons for their use is therefore welcome. The authors of the study under review had the opportunity to follow up the benzodiazepine of a sample of patients during their hospital stay in a Dutch academic hospital. The study is embedded in a larger trial, the WEsleep trial, to improve sleep quality in hospitals.

The authors studied the pre-admission use of benzodiazepines, including Z-drugs, the initiation and (dis)continuity of these medicines during hospitalisation and the use of these medicines at discharge. About 10% of the patients admitted to hospital used benzodiazepines/Z-drugs before hospitalisation, even more patients started with such a drug during hospitalisation, but the hospital doctors stopped their use in nearly 90% of them at discharge. The authors were alarmed about these results, especially since hospital doctors evaluated and changed benzodiazepine use in nearly none of the patients who used them before admission.

Apart from some minor problems (see # 3 ff.), I have two major concerns with this paper:

1. First of all, the design of the study is not appropriate to determine “whether prolonged use is primarily influenced by hospital-based care or by primary care” (lines 146 f.). Neither do we know how long the patient used these medicines before admission, nor do we know whether the patients will use (or their community doctors will prescribe) these medicines after discharge (see also # 11). So, the research question needs to be clarified in order not to raise false expectations or draw premature conclusions.

2. For me, one of the most important results in this study is the high number of patients who receive benzodiazepines/Z-drugs either from their primary care physician or in the hospital without (a documented) reason. The authors “hide” this result in the Supplementary part of the paper but it should get high visibility, in the Abstract, in the Results section as well as in the Discussion. Perhaps, it’s just these ‘secret reasons’ that could explain much of the attractiveness why doctors prescribe the medicines and patients ask for them.

3. I would like to ask the authors to check once more the literature on this subject. Just to mention two studies which had similar results and even similar design (at least the second one, that studied medication before admission, during hospitalisation and after discharge): https://pubmed.ncbi.nlm.nih.gov/32616945/; https://pubmed.ncbi.nlm.nih.gov/29202811/. These studies (and perhaps more of the same type), should be mentioned in the Introduction and should be used to compare the results of the new study in the Discussion.

4. The Study Design section in the Methods chapter (151 ff.) needs thorough revision. To date, it is more or less a description of the main trial (WEsleep) but has little to do with the present study. It is completely enough to mention the main trial in 1 sentence and with 1 reference. Instead, the authors should shortly describe what they did in the present study.

5. Similarly, lines 184 – 190 of the Data Collection section have little to do with the present study, but, again, highlight the main trial. Please, do not make reading unnecessary lengthy.

6. How did you obtain the information about prior use (193 ff.)? By admission letters? If so, please describe and give a note on how valid this information is.

7. I suppose that “discontinuation” at hospital discharge takes place in form of a discharge letter or recommendation for primary care doctors. Or do patients receive their medicines at discharge for a longer duration? Please describe this in more detail, especially if there are any peculiarities in the Netherlands system which readers should know.

8. Authors, do you think it may be useful to categorise the medicines under study in form of short-term versus long-term duration? If taken as sleeping pills, especially long-term medicine are considered to be dangerous.

9. The beginning of the Results section is difficult to read. Before you distinguish drug use between women and men, you should present the results for the whole sample. I would also recommend not to “jump” between the 3 times of data collection (line 224: home; line 226: hospital; line 229: home – really confusing).

10. Authors, perhaps you may once more think about your term “paradox” to describe the hospital doctors’ performance in drug management. Is it really a paradox or more or less a way to resolve a conflict-laden issue, i.e., psycho-drug management, without coming into conflict with the community colleagues and to guarantee a smooth running of the hospital, especially during nights, while trying to remove all traces at discharge?

11. You suppose that differences in continuation rates of benzodiazepine and Z-drugs prescriptions between your study and ref. 13 may be caused by differences in study design, patient samples and so on. I think, the main reason of this difference is that ref. 13 studies the real prescriptions in primary care practices while you consider only the drugs recommended by the hospital but not whether or not primary care doctors will follow their hospital colleagues (see also my comment # 1). If you agree, you should add this explanation.

12. Please shorten the Discussion. There are general remarks about sleep in hospitals (lines 340 ff.) or cooperation with primary care (352 ff. or 394 ff.). Quite nice but not supported by the results of your study and readers may have the feeling your main aim is to promote the main sleep trial and not the results of the present study.

13. Even if benzodiazepines for most patients were stopped at discharge, positive experience with these drugs during hospitalisation may create the desire or a need for these medications even after discharge so that patients may ask their doctor to prescribe them and, thus, promote long-term use (see, for example, https://pubmed.ncbi.nlm.nih.gov/31179613/). If you agree, you may shortly discuss this “knock-on effect”.

**Reviewer**	**2**
**Name**	**Keller, Michelle**
**Affiliation**	**Cedars-Sinai Medical Center, Medicine**
**Date**	**15-Dec-2025**
**COI**	**None**

This paper examines benzodiazepine use prior to admission, during admission, and at discharge as part of a secondary analysis of the WeSleep intervention. The paper is strong in that it examines the potential for hospitalization to lead to the incident prescribing of benzodiazepines. Several revisions would strengthen the manuscript, including: in the introduction, adding citations documenting long-term benzodiazepine use patterns (three references provided) and discussing barriers to inpatient deprescribing, particularly clinician resistance; in the methods, clarifying throughout that zolpidem and zopiclone are included (consider terminology like "sedative hypnotics" or "benzodiazepine receptor-agonists"), specifying the original WeSleep trial's pre-specified outcomes and whether benzodiazepine

discontinuation was included, defining "incident use" more precisely, and including data on PRN vs. scheduled prescribing if available; critically, addressing the absence of any analysis comparing intervention versus control groups despite WeSleep including physician education on benzodiazepine risks, either conduct statistical comparison of benzodiazepine outcomes between groups or explicitly frame this as exploratory/post-hoc analysis; and in the discussion, acknowledging practical limitations of inpatient deprescribing including withdrawal concerns and the difference between PRN and daily users, and exploring reasons for continuation at discharge (such as patient preferences) to better inform future interventions.

Detailed recommended revisions are below:

Introduction

1. This sentence “Despite their clinical usefulness in acute situations, such as during hospital admissions, benzodiazepines are often used for prolonged periods, contrary to guideline recommendations” needs a citation and could use further elaboration, for example, there is great research examining long-term benzo use.

a. Isacson D, Carsjö K, Bergman U, Blackburn JL. Long-term use of benzodiazepines in a Swedish community: an eight-year follow-up. J Clin Epidemiol. 1992 Apr;45(4):429-36. doi: 10.1016/0895-4356(92)90044-n. PMID: 1569439.

b. Isomura, K., Wang, X., Chang, Z. et al. Factors associated with long-term benzodiazepine and Z-drug use across the lifespan and 5-year temporal trajectories among incident users: a Swedish nationwide register-based study. Eur J Clin Pharmacol 79, 1091–1105 (2023). https://doi.org/10.1007/s00228-023-03515-2

c. Taipale H, Särkilä H, Tanskanen A, et al. Incidence of and Characteristics Associated With Long-term Benzodiazepine Use in Finland. JAMA Netw Open. 2020;3(10):e2019029. doi:10.1001/jamanetworkopen.2020.19029

2. There are also myriad other factors that prevent clinicians from deprescribing benzodiazepines during hospitalization, including resistance from inpatient clinicians regarding starting the process of deprescribing (many see it as an outpatient issue), which should be raised in the introduction, see the following paper:

a. Keller MS, Carrascoza-Bolanos J, Breda K, Kim LY, Kennelty KA, Leang DW, Murry LT, Nuckols TK, Schnipper JL, Pevnick JM. Identifying barriers and facilitators to deprescribing benzodiazepines and sedative hypnotics in the hospital setting using the Theoretical Domains Framework and the Capability, Opportunity, Motivation and Behaviour (COM-B) Model: a qualitative study. BMJ Open. 2023 Feb 22;13(2):e066234.

Methods

1. The authors note that they included zolpidem and zopiclone in their analysis, I would recommend revising the introduction, abstract, and title to note that these medications are

included. Others in the literature have termed these “sedative hypnotics” or “benzodiazepine receptor-agonists.”

2. The authors should include the pre-specified primary and secondary outcomes of the clinical trial were and how they were measured. It would also be important to note if benzodiazepine discontinuation or deprescription (reducing the dose) was pre-specified as part of the clinical trial and if there were any hypotheses associated with benzos as part of this work.

3. How was “incident use” defined? For example, if the patient had taken benzodiazepines 3 months ago but wasn’t currently taking them, was this defined as incident use?

4. There is no discussion of examining the effect of the WeSleep intervention on benzodiazepine use, which is puzzling, considering the intervention did include physician education on the risks of benzodiazepine use. If the benzodiazepine use outcome was not pre-specified, the authors could note that this is a post-hoc or exploratory outcome and do some of the statistical analyses examining the effect of the intervention on the outcome. For example, it appeared that benzodiazepine initiation was lower in the intervention group? If there were no meaningful differences between the intervention and control group, then the authors should do the proper statistical analysis and make the point that this wasn’t the primary outcome, and then note that a different intervention is needed to reduce benzo prescribing.

5. It would be helpful (if the data are available) whether the benzodiazepines were prescribed PRN or scheduled.

Discussion

1. The authors make the point that there may be a missed opportunity to begin deprescribing in patients with long-term use, but hospitalizations are often too short to do meaningful reductions. Deprescribing in the hospital could be helpful among patients who are taking them PRN and thus not taking them daily, but may be much more difficult among patients who are taking them every night due to concerns about withdrawal symptoms.

2. The authors make an excellent point about the potential harms of initiating benzodiazepines during hospitalization and continuing the medications at discharge. Understanding why this is happening (patient preference?) would be helpful to curbing this incident use and is an area for future research.

**Reviewer**	**3**
**Name**	**Young, Alan**
**Affiliation**	**Department of Respiratory and Sleep Medicine at Eastern Health**
**Date**	**16-Dec-2025**

COI **None**

This is an appropriately designed study, with important findings that are clinically relevant. The results are presented clearly and the manuscript is well written.

The study is a sub-analysis of the WEsleep trial, a cluster RCT encompassing 6 medical and 6 surgical wards in a single centre (2 locations). The key findings are that prior BZD usage changed very little during admission, whilst newly prescribed BZD usage was mostly ceased at discharge (88%). New BZD use was commoner in surgical patients and women had higher rates of BZD prescription prior and during hospital admission.

Looking at Table 1, 'BZD newly initiated in hospital' group (n=107), there is a lower proportion for intervention group than the standard care group (42.1 vs 57.9%).

Does this suggest there was less 'new' BZD prescribing in the intervention group?

Could this be explained by BZD prescribing education provided to the intervention group or that the sleep intervention itself (eg reduced interruptions) improved sleep resulting in lower need for BZD prescription? Could the authors please clarify and address in the results and discussion section. (I do note overall BZD prescribing rates did not differ between the intervention and standard group in the WeSleep trial).

VERSION 1 - AUTHOR RESPONSE

Reviewer: 1
Prof. Wolfgang Himmel, University Medical Center Göttingen
Comments to the Author:
The use of benzodiazepines in hospitals and in the community is still very high—despite warnings against their use, especially their long-term use. Any information about their prescribing patterns and possible reasons for their use is therefore welcome. The authors of the study under review had the opportunity to follow up the benzodiazepine of a sample of patients during their hospital stay in a Dutch academic hospital. The study is embedded in a larger trial, the WEsleep trial, to improve sleep quality in hospitals.

The authors studied the pre-admission use of benzodiazepines, including Z-drugs, the initiation and (dis)continuity of these medicines during hospitalisation and the use of these medicines at discharge. About 10% of the patients admitted to hospital used benzodiazepines/Z-drugs before hospitalisation, even more patients started with such a drug during hospitalisation, but the hospital doctors stopped their use in nearly 90% of them at discharge. The authors were alarmed about these results, especially since hospital doctors evaluated and changed benzodiazepine use in nearly none of the patients who used them before admission.

Apart from some minor problems (see # 3 ff.), I have two major concerns with this paper:

1. First of all, the design of the study is not appropriate to determine "whether prolonged use is primarily influenced by hospital-based care or by primary care" (lines 146 f.). Neither do we know how long the patient used these medicines before admission, nor do we know whether the patients

will use (or their community doctors will prescribe) these medicines after discharge (see also # 11). So, the research question needs to be clarified in order not to raise false expectations or draw premature conclusions.

Thank you for your comments. We agree that the original wording could raise expectations regarding the assessment of prolonged benzodiazepine use and the relative contribution of hospital-based versus primary care prescribing, which cannot be determined with the available data. We have therefore revised the study aim in the manuscript to more accurately reflect the study design and analyses performed [153-158].

2. For me, one of the most important results in this study is the high number of patients who receive benzodiazepines/Z-drugs either from their primary care physician or in the hospital without (a documented) reason. The authors "hide" this result in the Supplementary part of the paper but it should get high visibility, in the Abstract, in the Results section as well as in the Discussion. Perhaps, it's just these 'secret reasons' that could explain much of the attractiveness why doctors prescribe the medicines and patients ask for them.

We agree that the high proportion of benzodiazepine/Z-drug prescriptions without a documented indication is a key finding. The manuscript has been revised to give this result greater visibility in both the Abstract and Discussion sections [397-401].

3. I would like to ask the authors to check once more the literature on this subject. Just to mention two studies which had similar results and even similar design (at least the second one, that studied medication before admission, during hospitalisation and after discharge): https://pubmed.ncbi.nlm.nih.gov/32616945/; https://pubmed.ncbi.nlm.nih.gov/29202811/. These studies (and perhaps more of the same type), should be mentioned in the Introduction and should be used to compare the results of the new study in the Discussion.

We have added a citation to compare our results in the Discussion [482-485].

4. The Study Design section in the Methods chapter (151 ff.) needs thorough revision. To date, it is more or less a description of the main trial (WEsleep) but has little to do with the present study. It is completely enough to mention the main trial in 1 sentence and with 1 reference. Instead, the authors should shortly describe what they did in the present study.

We have substantially revised and shortened the Methods section to improve clarity and focus on the present sub-analysis. The description of the WEsleep trial has been reduced in the Study Design section.

5. Similarly, lines 184 – 190 of the Data Collection section have little to do with the present study, but, again, highlight the main trial. Please, do not make reading unnecessary lengthy.

The Data Collection section has been streamlined by removing trial-specific details that are not directly relevant to this analysis. Both sections now explicitly describe only the design, patient population, and data extraction procedures pertinent to the current study [225-260].

6. How did you obtain the information about prior use (193 ff.)? By admission letters? If so, please describe and give a note on how valid this information is.

Information on benzodiazepine use prior to hospital admission was obtained through a structured medication reconciliation process performed by the hospital pharmacy as part of routine care. This process involves verification of the patient's home medication using multiple sources, including community pharmacy dispensing records and patient interviews. While minor inaccuracies cannot be completely excluded, this approach is currently considered the most reliable method available for documenting pre-admission medication use in clinical practice. We have added a description of how information on benzodiazepine use prior to hospital admission was obtained to the Methods section of the manuscript [246-251]].

7. I suppose that "discontinuation" at hospital discharge takes place in form of a discharge letter or recommendation for primary care doctors. Or do patients receive their medicines at discharge for a longer duration? Please describe this in more detail, especially if there are any peculiarities in the Netherlands system which readers should know.

In the Netherlands, medication changes at hospital discharge, including discontinuation of benzodiazepines, are implemented in the hospital prescribing system and communicated electronically to the patient's community pharmacy as part of the discharge medication reconciliation process. In addition, medication changes and recommendations are documented in the discharge letter sent to the general practitioner. Patients do not routinely receive hospital-issued medication for long-term use after discharge; ongoing prescribing is coordinated through primary care. We have added a description of this discharge process to the Methods section to clarify how discontinuation at discharge was defined and communicated [257-260].

8. Authors, do you think it may be useful to categorise the medicines under study in form of short-term versus long-term duration? If taken as sleeping pills, especially long-term medicine are considered to be dangerous.

We agree that distinguishing between short-term and long-term benzodiazepine use is clinically relevant, particularly given the risks associated with long-term use. As described in the Limitations section, the available data unfortunately did not allow reliable classification of duration of use before admission or after discharge. We therefore did not further categorize prescriptions by duration.

9. The beginning of the Results section is difficult to read. Before you distinguish drug use between women and men, you should present the results for the whole sample. I would also recommend not to "jump" between the 3 times of data collection (line 224: home; line 226: hospital; line 229: home – really confusing).

We have revised the beginning of the Results section to first present findings for the total study population before reporting subgroup differences. In addition, the Results are now structured consistently by time point, avoiding unnecessary switching between home use, in-hospital use, and discharge [311-318].

10. Authors, perhaps you may once more think about your term "paradox" to describe the hospital doctors' performance in drug management. Is it really a paradox or more or less a way to resolve a conflict-laden issue, i.e., psycho-drug management, without coming into conflict with the community colleagues and to guarantee a smooth running of the hospital, especially during nights, while trying to remove all traces at discharge?

We agree that the term “paradox” may suggest an unintended value judgment. We have therefore revised the Discussion to remove this term and now describe two key findings in inpatient benzodiazepine management separately: the predominantly short-term nature of newly initiated prescriptions and the limited reassessment of pre-existing benzodiazepine use during hospitalization. This revised wording avoids interpretative framing and better reflects the observational nature of our data.

11. You suppose that differences in continuation rates of benzodiazepine and Z-drugs prescriptions between your study and ref. 13 may be caused by differences in study design, patient samples and so on. I think, the main reason of this difference is that ref. 13 studies the real prescriptions in primary care practices while you consider only the drugs recommended by the hospital but not whether or not primary care doctors will follow their hospital colleagues (see also my comment # 1). If you agree, you should add this explanation.

We have revised the Discussion to explicitly state that our study evaluates prescribing at hospital discharge, whereas the referenced study assessed subsequent prescribing in primary care.

12. Please shorten the Discussion. There are general remarks about sleep in hospitals (lines 340 ff.) or cooperation with primary care (352 ff. or 394 ff.). Quite nice but not supported by the results of your study and readers may have the feeling your main aim is to promote the main sleep trial and not the results of the present study.

We have shortened the Discussion by removing general statements on sleep in hospitals and collaboration with primary care that were not directly supported by the results of the present study, thereby refocusing the Discussion on the key findings of this analysis.

13. Even if benzodiazepines for most patients were stopped at discharge, positive experience with these drugs during hospitalisation may create the desire or a need for these medications even after discharge so that patients may ask their doctor to prescribe them and, thus, promote long-term use (see, for example, https://pubmed.ncbi.nlm.nih.gov/31179613/). If you agree, you may shortly discuss this “knock-on effect”.

We have added this to the discussion section [409-412].

Reviewer: 2
Dr. Michelle Keller, Cedars-Sinai Medical Center
Comments to the Author:
This paper examines benzodiazepine use prior to admission, during admission, and at discharge as part of a secondary analysis of the WeSleep intervention. The paper is strong in that it examines the potential for hospitalization to lead to the incident prescribing of benzodiazepines. Several revisions would strengthen the manuscript, including: in the introduction, adding citations documenting long-term benzodiazepine use patterns (three references provided) and discussing barriers to inpatient deprescribing, particularly clinician resistance; in the methods, clarifying throughout that zolpidem and zopiclone are included (consider terminology like "sedative hypnotics" or "benzodiazepine receptor-agonists"), specifying the original WeSleep trial's pre-specified outcomes and whether benzodiazepine discontinuation was included, defining "incident use" more precisely, and including data on PRN vs. scheduled prescribing if available; critically, addressing the absence of any analysis comparing intervention versus control groups despite WeSleep including physician education on

benzodiazepine risks, either conduct statistical comparison of benzodiazepine outcomes between groups or explicitly frame this as exploratory/post-hoc analysis; and in the discussion, acknowledging practical limitations of inpatient deprescribing including withdrawal concerns and the difference between PRN and daily users, and exploring reasons for continuation at discharge (such as patient preferences) to better inform future interventions.

Detailed recommended revisions are below:

Introduction

1. This sentence “Despite their clinical usefulness in acute situations, such as during hospital admissions, benzodiazepines are often used for prolonged periods, contrary to guideline recommendations” needs a citation and could use further elaboration, for example, there is great research examining long-term benzo use.

a. Isacson D, Carsjö K, Bergman U, Blackburn JL. Long-term use of benzodiazepines in a Swedish community: an eight-year follow-up. J Clin Epidemiol. 1992 Apr;45(4):429-36. doi: 10.1016/0895-4356(92)90044-n. PMID: 1569439.

b. Isomura, K., Wang, X., Chang, Z. et al. Factors associated with long-term benzodiazepine and Z-drug use across the lifespan and 5-year temporal trajectories among incident users: a Swedish nationwide register-based study. Eur J Clin Pharmacol 79, 1091–1105 (2023). https://doi.org/10.1007/s00228-023-03515-2

c. Taipale H, Särkilä H, Tanskanen A, et al. Incidence of and Characteristics Associated With Long-term Benzodiazepine Use in Finland. JAMA Netw Open. 2020;3(10):e2019029. doi:10.1001/jamanetworkopen.2020.19029

Thank you for your comments. We have added a citation in the introduction section.

2. There are also myriad other factors that prevent clinicians from deprescribing benzodiazepines during hospitalization, including resistance from inpatient clinicians regarding starting the process of deprescribing (many see it as an outpatient issue), which should be raised in the introduction, see the following paper:

a. Keller MS, Carrascoza-Bolanos J, Breda K, Kim LY, Kennelty KA, Leang DW, Murry LT, Nuckols TK, Schnipper JL, Pevnick JM. Identifying barriers and facilitators to deprescribing benzodiazepines and sedative hypnotics in the hospital setting using the Theoretical Domains Framework and the Capability, Opportunity, Motivation and Behaviour (COM-B) Model: a qualitative study. BMJ Open. 2023 Feb 22;13(2):e066234.

We have added this citation in the introduction section.

Methods

1. The authors note that they included zolpidem and zopiclone in their analysis, I would recommend revising the introduction, abstract, and title to note that these medications are included. Others in the literature have termed these “sedative hypnotics” or “benzodiazepine receptor-agonists.”

The introduction, abstract, and title have been revised to explicitly note the inclusion of zolpidem and zopiclone and to describe these agents as benzodiazepine receptor agonists, consistent with terminology used in the literature.

2. The authors should include the pre-specified primary and secondary outcomes of the clinical trial were and how they were measured. It would also be important to note if benzodiazepine discontinuation or deprescription (reducing the dose) was pre-specified as part of the clinical trial and if there were any hypotheses associated with benzos as part of this work.

Use of sleep medication was pre-specified as a secondary outcome of the WEsleep trial. However, benzodiazepine or benzodiazepine receptor agonist discontinuation or dose reduction was not defined as a distinct outcome, nor were a priori hypotheses formulated specifically regarding deprescribing. The present sub-analysis therefore extends the pre-specified outcome of sleep medication use by providing a more detailed, exploratory examination of initiation, continuation, and discontinuation patterns during hospitalization and at discharge. We have added the primary and secondary outcomes to the methods section [164-168].

3. How was “incident use” defined? For example, if the patient had taken benzodiazepines 3 months ago but wasn’t currently taking them, was this defined as incident use?

To avoid ambiguity, we have revised the Methods and no longer use the term “incident use.” In the revised manuscript, we explicitly describe patients as either having pre-existing benzodiazepine or benzodiazepine receptor agonist use at admission or having medication newly initiated during hospitalization. Patients who were not using these medications at the time of admission, including those with prior use in the past but no active prescription, were classified as not using at admission. This clarification has been incorporated throughout the manuscript.

4. There is no discussion of examining the effect of the WeSleep intervention on benzodiazepine use, which is puzzling, considering the intervention did include physician education on the risks of benzodiazepine use. If the benzodiazepine use outcome was not pre-specified, the authors could note that this is a post-hoc or exploratory outcome and do some of the statistical analyses examining the effect of the intervention on the outcome. For example, it appeared that benzodiazepine initiation was lower in the intervention group? If there were no meaningful differences between the intervention and control group, then the authors should do the proper statistical analysis and make the point that this wasn’t the primary outcome, and then note that a different intervention is needed to reduce benzo prescribing.

Thank you for this comment. The WEsleep intervention was primarily designed to improve sleep quality during hospitalization and was not intended to directly change benzodiazepine prescribing behavior. Although physician education on benzodiazepine risks was included and use of sleep medication was a pre-specified secondary outcome, no significant differences in prescribing were observed between intervention and control groups in the main trial. Benzodiazepine initiation or deprescribing was not a primary outcome, and no a priori hypotheses were formulated. Given this, and the absence of meaningful between-group differences, we did not perform additional post-hoc intervention-effect analyses in this sub-analysis, which focuses on describing prescribing patterns rather than evaluating intervention effects.

5. It would be helpful (if the data are available) whether the benzodiazepines were prescribed PRN or scheduled.

Unfortunately, this data is not available.

Discussion

1. The authors make the point that there may be a missed opportunity to begin deprescribing in patients with long-term use, but hospitalizations are often too short to do meaningful reductions. Deprescribing in the hospital could be helpful among patients who are taking them PRN and thus not taking them daily, but may be much more difficult among patients who are taking them every night due to concerns about withdrawal symptoms.

We agree that the feasibility of deprescribing during hospitalization depends on the pattern and duration of benzodiazepine use. We have revised the Discussion to clarify that while hospitalization may offer an opportunity for reassessment and potential discontinuation among patients using benzodiazepines on a PRN basis, meaningful dose reduction or cessation among patients with daily or long-term use may be challenging during typically short hospital stays due to concerns about withdrawal symptoms. We now emphasize that, particularly for chronic users, hospitalization may primarily serve as a moment for review, documentation, and planning of deprescribing after discharge rather than for complete inpatient tapering [491-498].

2. The authors make an excellent point about the potential harms of initiating benzodiazepines during hospitalization and continuing the medications at discharge. Understanding why this is happening (patient preference?) would be helpful to curbing this incident use and is an area for future research.

We have revised the Discussion to further elaborate on the potential harms of initiating benzodiazepines during hospitalization and continuing them at discharge.

Reviewer: 3

Dr. Alan Young, Department of Respiratory and Sleep Medicine at Eastern Health, Monash University Eastern Health Clinical School

Comments to the Author:

This is an appropriately designed study, with important findings that are clinically relevant. The results are presented clearly and the manuscript is well written.

The study is a sub-analysis of the WEsleep trial, a cluster RCT encompassing 6 medical and 6 surgical wards in a single centre (2 locations). The key findings are that prior BZD usage changed very little during admission, whilst newly prescribed BZD usage was mostly ceased at discharge (88%). New BZD use was commoner in surgical patients and women had higher rates of BZD prescription prior and during hospital admission.

Looking at Table 1, ‘BZD newly initiated in hospital’ group (n=107), there is a lower proportion for intervention group than the standard care group (42.1 vs 57.9%).

Does this suggest there was less ‘new’ BZD prescribing in the intervention group?

Could this be explained by BZD prescribing education provided to the intervention group or that the sleep intervention itself (eg reduced interruptions) improved sleep resulting in lower need for BZD prescription? Could the authors please clarify and address in the results and discussion section. (I do note overall BZD prescribing rates did not differ between the intervention and standard group in the WeSleep trial).

Thank you for this observation. Although the proportion of patients with newly initiated benzodiazepines appears lower in the intervention group in Table 1, the main WEsleep trial found no statistically significant differences in overall benzodiazepine prescribing between the intervention and standard care groups. As such, the observed difference in proportions in this sub-analysis should be interpreted cautiously and may reflect random variation rather than an intervention effect. While the intervention included physician education on benzodiazepine risks and measures aimed at improving sleep quality, the primary aim of the WEsleep trial was to improve sleep quality, and no conclusions regarding reductions in benzodiazepine initiation can be drawn from this secondary analysis.

VERSION 2 - REVIEW

Reviewer 1

Name Himmel, Wolfgang

Affiliation University Medical Center Göttingen, Department of General Practice/Family Medicine

Date 19-Jan-2026

COI

Thank you, authors, for considering my (our) recommendations in the new version of your manuscript. I have only some minor comments and hope you find a way to incorporate them in a final version.

1. While the term “paradox” is no longer used at the end of the paper in the new version, the term is still present in the Conclusions section of the Abstract. You should also change this section accordingly.

2. You now mention the often missing indication for the use of benzodiazepines in the Conclusions section of the Abstract but this is for readers difficult to understand as long as you do not mention this aspect also in the Results section of the Abstract. In spite of the word limit in the Abstract, it should be possible to include a further sentence in the Abstract where you may shortly mention that about half of the of the benzodiazepines before hospital admission and about one-third in the hospital were prescribed without a documented indication.

3. You conclude that the frequent absence of documented indications limits the evaluation of the prescribing process during hospitalization (lines 77 f., 307 ff.). Perhaps you may add that your findings are a plea for both doctors, hospital and community doctors, to prescribe these medicines more carefully and only for those indications that are in line with guidelines.

4. I would recommend to include a table, combined from Supplement tables 3 and 6, in the full text - and not only in the Supplement section so that readers are directly informed in the

main text and fully aware of the (missing) indications for benzodiazepines before hospital admission and in the hospital. I'm sure, BMJOpen allows you to include a further table in the main body of the text.

5. Thank you, authors that you incorporated the idea that positive hospital experience with benzodiazepines and Z-drugs may influence patient expectations to receive them after discharge (lines 318 ff.). I would appreciate if this new aspect in your paper is accordingly referenced (https://pubmed.ncbi.nlm.nih.gov/31179613) but since I am co-author of the study, I don't want to come into a conflict of interest. So, I would like to make sure that I accept your paper independently of whether or not you cite this paper.

**Reviewer**	**3**
**Name**	**Young, Alan**
**Affiliation**	**Department of Respiratory and Sleep Medicine at Eastern Health**
**Date**	**19-Jan-2026**
**COI**	

Thank you, I am satisfied with the response to my query.

VERSION 2 - AUTHOR RESPONSE

Reviewer: 1
Prof. Wolfgang Himmel, University Medical Center Göttingen
Comments to the Author:
Thank you, authors, for considering my (our) recommendations in the new version of your manuscript. I have only some minor comments and hope you find a way to incorporate them in a final version.

1. While the term "paradox" is no longer used at the end of the paper in the new version, the term is still present in the Conclusions section of the Abstract. You should also change this section accordingly.

Thank you for your comment. The abstract conclusion has been revised to improve clarity and better reflect the key findings of the study.

2. You now mention the often missing indication for the use of benzodiazepines in the Conclusions section of the Abstract but this is for readers difficult to understand as long as you do not mention this aspect also in the Results section of the Abstract. In spite of the word limit in the Abstract, it should be possible to include a further sentence in the Abstract where you may shortly mention that about half of the of the benzodiazepines before hospital admission and about one-third in the hospital were prescribed without a documented indication.

We have revised the Results section of the Abstract to include information on the proportion of benzodiazepine prescriptions without a documented indication, in line with the Conclusions section.

3. You conclude that the frequent absence of documented indications limits the evaluation of the prescribing process during hospitalization (lines 77 f., 307 ff.). Perhaps you may add that your findings are a plea for both doctors, hospital and community doctors, to prescribe these medicines more carefully and only for those indications that are in line with guidelines.

Thank you for this suggestion. We have revised the Conclusions section to explicitly emphasize the need for more cautious, guideline-adherent benzodiazepine prescribing by both hospital-based and community physicians.

4. I would recommend to include a table, combined from Supplement tables 3 and 6, in the full text - and not only in the Supplement section so that readers are directly informed in the main text and fully aware of the (missing) indications for benzodiazepines before hospital admission and in the hospital. I'm sure, BMJOpen allows you to include a further table in the main body of the text.

We have added the Table.

5. Thank you, authors that you incorporated the idea that positive hospital experience with benzodiazepines and Z-drugs may influence patient expectations to receive them after discharge (lines 318 ff.). I would appreciate if this new aspect in your paper is accordingly referenced (https://pubmed.ncbi.nlm.nih.gov/31179613) but since I am co-author of the study, I don't want to come into a conflict of interest. So, I would like to make sure that I accept your paper independently of whether or not you cite this paper.

We have added this citation.
